# Kinetically stabilized 1,3-diarylisobenzofurans and the possibility of preparing large, persistent isoacenofurans with unusually small HOMO–LUMO gaps

**DOI:** 10.3762/bjoc.20.97

**Published:** 2024-05-17

**Authors:** Qian Liu, Glen P Miller

**Affiliations:** 1 Department of Chemistry, University of New Hampshire, 23 Academic Way, Durham, New Hampshire 03864-3598, USA

**Keywords:** acene, DFT calculation, highly delocalized π-system, isoacenofuran, isobenzofuran, kinetically stabilized, organic semiconductor, small HOMO–LUMO gap, synthesis

## Abstract

DFT calculations demonstrate that an isoacenofuran of any size possesses a smaller HOMO–LUMO gap than the corresponding acene bearing an isoelectronic π-system (i.e., the same total number of rings). Isoacenofurans show limited stability due in part to the highly reactive 1,3-carbons of the furan ring. Both 1,3-dimesitylisobenzofuran and 1,3-di(2’,4’,6’-triethylphenyl)isobenzofuran, each bearing sterically congesting *ortho*-alkyl groups on their phenyl substituents, have been synthesized and shown to adopt non-planar conformations with the *ortho*-alkyl groups located above and below the most reactive 1,3-carbons of the furan ring. These bulky substituents provide a strong measure of kinetic stabilization. Thus, 1,3-dimesitylisobenzofuran and 1,3-di(2’,4’,6’-triethylphenyl)isobenzofuran are significantly less reactive than 1,3-diphenylisobenzofuran toward the strong dienophiles DMAD and acrylonitrile. The insights gained here suggest that the synthesis of large, persistent, kinetically stabilized isoacenofurans with unusually small HOMO–LUMO gaps is achievable. As such, these molecules deserve increased attention as potential p-type organic semiconductors.

## Introduction

**Figure 1:**
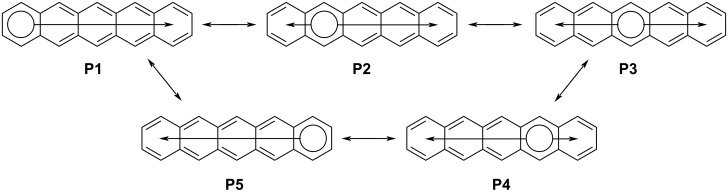
Neutral, closed-shell resonance forms for pentacene highlighting Clar aromatic sextets (see [1]) and the degree to which the remaining π-electrons are extensively delocalized, or not.

Acenes are composed of linearly annellated benzene rings. Compared to their non-linearly annellated isomers, acenes possess smaller HOMO–LUMO gaps. This is attributed to their novel electronic structures which manifest that no more than one benzene ring can be drawn with a full aromatic sextet in any neutral, closed-shell resonance form (Figure 1) [1]. One can view an aromatic sextet in any one resonance form as a set of six π-electrons that are localized to one ring whereas other π-electrons are delocalized over the remaining π-system. We associate larger, more highly delocalized π-systems with smaller HOMO–LUMO gaps.

**Figure 2:**
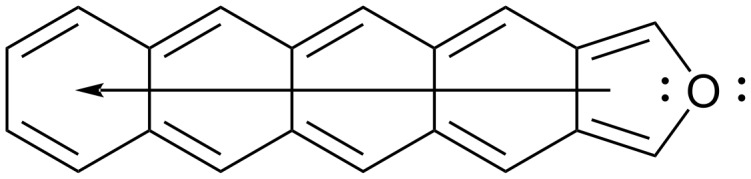
The only neutral, closed-shell resonance form for 5-ring isotetracenofuran with its highly delocalized π-system.

Isoacenofurans are composed of linearly annellated benzene rings that terminate with a furan ring. Isoacenofurans and acenes possess isoelectronic π-systems when the total number of rings is the same. Unlike acenes, none of the 6-membered rings in an isoacenofuran possess an aromatic sextet in any neutral, closed-shell resonance form (Figure 2). Thus, compared to the corresponding acene, an isoacenofuran could possess a more highly delocalized π-system and an even smaller HOMO–LUMO gap. For example, consider pentacene and isotetracenofuran. Pentacene can be drawn in several neutral, closed-shell resonance forms (Figure 1) in which any one of the five 6-membered rings possess an aromatic sextet. If the aromatic sextet is located in a terminal ring of the molecule, as in resonance forms **P1** and **P5** of Figure 1, then the remaining π-electrons are delocalized over four rings. However, calculations indicate that the center ring of pentacene and other acenes is the most aromatic [2] and therefore resonance form **P3** of Figure 1 is most significant.

By contrast, isotetracenofuran and other isoacenofurans can only be drawn in one neutral, closed-shell resonance form in which only the furan ring possesses an aromatic sextet (Figure 2). The remaining π-electrons of isotetracenofuran are fully delocalized over the remaining four rings. This analysis suggests that all isoacenofurans should possess a more highly delocalized π-system than their acene counterparts, and potentially with smaller HOMO–LUMO gaps. However, a systematic study of HOMO–LUMO gaps for isoelectronic acene–isoacenofuran pairs has not been published. Should such a study reveal unusually small HOMO–LUMO gaps for isoacenofurans, we would consider persistent versions of these molecules to be a highly interesting class of organic semiconductors.

Hamura and co-workers pioneered the synthesis of large isoacenofurans. They prepared a pair of 1,3-diarylisoanthracenofurans [3] and a pair of 1,3-diphenethynylisoanthracenofurans [4], and impressively utilized the latter as intermediates for the synthesis of stabilized hexacenes. Their beautiful work documented the lack of stability associated with isoanthracenofurans. Thus, their 1,3-diarylisoanthracenofurans rapidly oxidized in solution to form endoperoxides [3]. These compounds persisted longer in the solid state, but clearly the lack of stability and persistence associated with isoacenofurans is of great concern if these molecules are to be utilized as organic semiconductors. Likewise, Hamura and co-workers’ 1,3-diphenethynylisobenzofurans were fleeting intermediates that could not be isolated, but were instead trapped in situ by a suitable dienophile [4].

Large acenes are prone to photooxidation, especially when dissolved in solution while exposed to ambient light and air. They sensitize singlet oxygen formation, and the same is expected from large isoacenofurans. Indeed, the endoperoxides observed by Hamura and co-workers [3] confirm this expectation. We previously studied substituent effects in acenes and reported that several substituents promote photooxidative resistance in pentacenes [5] and larger acenes including heptacene [6] and nonacene [7]. One or more substituents that promote photooxidative resistance by quenching singlet oxygen could be utilized on isoacenofurans, too. Additionally, the 1,3-carbons of the furan ring in isoacenofurans are highly reactive towards dienophiles, including singlet oxygen. Thus, we believe that the design of large, persistent isoaceneofurans should include multiple substituents that provide photooxidative resistance, especially along the acene-like segment, plus a strong measure of kinetic stabilization at the 1,3-carbons of the furan ring.

As described here, there are compelling reasons to contemplate the synthesis of large, persistent isoacenofurans with unusually small HOMO–LUMO gaps. In this work, we probe several aspects of this challenge. First, we utilize a DFT method to calculate HOMO–LUMO gaps associated with several isoacenofurans and compare them to the calculated HOMO–LUMO gaps of their isoelectronic acenes. Second, we synthesized two 1,3-diarylisobenzofurans that provide steric resistance to the most reactive 1,3-carbons of the furan ring. A combination of experimental and computational studies clarifies the impacts of these sterically congesting substituents on each molecule’s electronic structure. We further studied the reaction rates of 1,3-diarylisobenzofurans with the strong dienophiles dimethyl acetelyenedicarboxylate (DMAD) and acrylonitrile. We conclude that the synthesis of large, persistent, kinetically stabilized isoacenofurans with unusually small HOMO–LUMO gaps is an achievable goal.

## Results and Discussion

### Computational HOMO–LUMO gaps for isoacenofurans and comparable acenes

**Figure 3:**
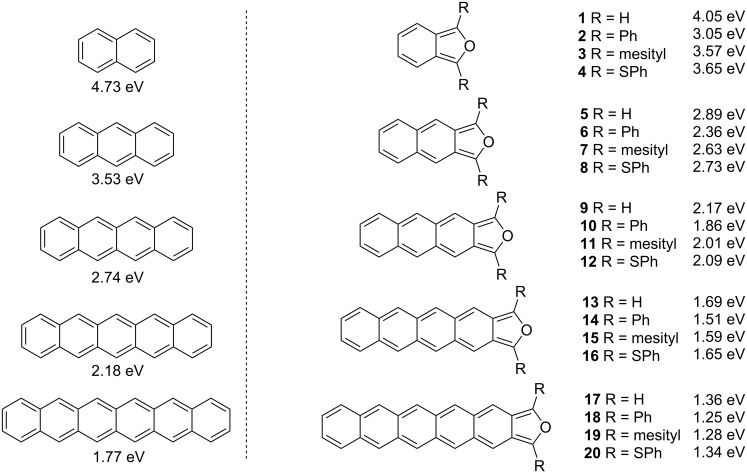
DFT calculated HOMO–LUMO gaps of acenes and isoacenofurans performed at the B3LYP/6-311+G(d,p)//B3LYP/6-31G(d) level of theory using Spartan ’20 [8].

**Figure 4:**
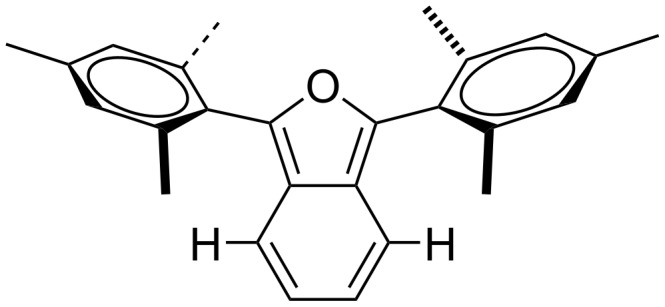
A structural rendering of 1,3-dimesitylisobenzofuran showing the requirement for non-planar mesityl groups in order to avoid steric repulsion between *ortho*-methyl groups and the nearest benzo hydrogen atoms.

We studied the HOMO–LUMO gaps associated with acenes and isoacenofurans (Figure 3) using a DFT method that has proven reliable for large acenes both in terms of absolute values and trends. In all cases, isoacenofurans possess a smaller HOMO–LUMO gap than the corresponding acene with an isoelectronic π-system. For example, naphthalene and isobenzofuran (**1**) both possess 10 π-electrons and are calculated to have HOMO–LUMO gaps of 4.73 and 4.05 eV, respectively. The incorporation of 1,3-diphenyl substituents (compound **2**) or 1,3-diphenylthio substituents (compound **4**) lowers the HOMO–LUMO gaps of the corresponding isobenzofurans to 3.05 and 3.65 eV, respectively. These groups provide for expanded delocalization of π-electron density outside the isobenzofuran core. Phenylthio substituents have also been shown to impart considerable photooxidative resistance to pentacene [5]. The incorporation of 1,3-dimesityl substituents (compound **3**) also provides for modest lowering of the HOMO–LUMO gap despite the non-planar geometry that these groups must adopt (Figure 4). We are keenly interested in preparing isoacenofurans with small HOMO–LUMO gaps. The non-planar geometry of mesityl and similar groups additionally provides for enhanced steric congestion at the most reactive 1,3-carbons of the furan ring (vide infra), and this could prove to be an important design strategy for large, persistent isoacenofurans.

The trend continues for the entire acene series calculated, from anthracene to hexacene (Figure 3). Thus, pentacene, a benchmark organic semiconductor, has a calculated HOMO–LUMO gap of 2.18 eV, nearly 0.5 eV greater than that of isotetracenofuran (**13**) with an isoelectronic π-system (22 π-electrons each). The incorporation of phenylthio groups (compound **16**) further lowers the HOMO–LUMO gap to 1.65 eV. Even in the presence of sterically congesting, non-planar 1,3-dimesityl groups, the corresponding isotetracenofuran (**15**) possesses an unusually small HOMO–LUMO gap of 1.59 eV.

Among the isoacenofurans studied here, the smallest calculated HOMO–LUMO gaps are observed in the isopentacenofuran series (compounds **17**–**20**). They possess HOMO–LUMO gaps between 1.25 and 1.36 eV, well below the 1.77 eV value calculated for hexacene. These Spartan ’20 [8] calculations confirm our expectation of a more highly delocalized π-system in any isoacenofuran (Figure 2) compared to the corresponding acene with isoelectronic π-system (Figure 1). As such, isoacenofurans represent a highly interesting class of molecules that, although largely ignored in the literature, deserve increased attention as organic semiconductors with unusually small HOMO–LUMO gaps.

### Synthesis of two 1,3-diarylisobenzofurans with sterically congesting substituents

**Scheme 1:**
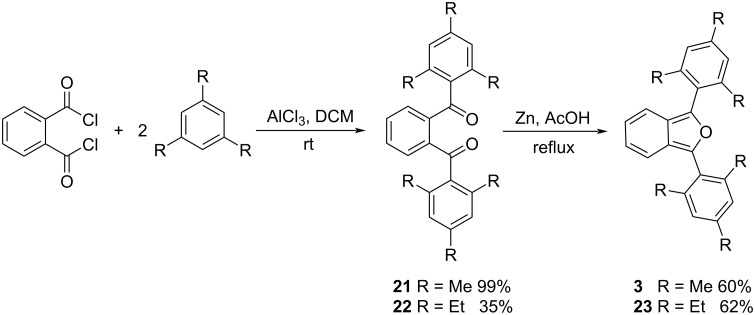
Synthesis of 1,3-diarylisobenzofurans **3** and **23**.

In order to study the impacts of sterically congesting substituents at the 1,3-carbons of the furan ring, we synthesized two 1,3-diarylisobenzofurans (compounds **3** and **23**, Scheme 1). Combined with commercially available **2**, these compounds provide varying degrees of steric congestion to the highly reactive 1,3-carbons on the furan ring. Thus, phthaloyl chloride was reacted with mesitylene or 1,3,5-triethylbenzene to produce the corresponding diketones **21** and **22**. Each diketone was in turn reacted with zinc metal in glacial acetic acid [9] to afford the corresponding 1,3-diarylisobenzofurans **3** and **23**. The latter reductions in the presence of zinc likely proceed through the corresponding ketols which are known to undergo ring closure in acidic solution [10–11]. These syntheses utilizing mesitylene or 1,3,5-triethylbenzene are considerably simpler than other approaches that would place sterically congesting groups only at the *ortho* positions of the 1,3-diaryl substituents. Compounds **3** and **23** contain an additional *para* substituent that serves no particular purpose but is innocuous.

The syntheses of compounds **24** and **25** with 2’,4’,6’-triisopropylphenyl and 2’,4’,6’-tri-*tert*-butylphenyl substituents, respectively, were also attempted but without success. In these cases, the sterically congesting *ortho* isopropyl and *tert*-butyl groups stymie the Friedel–Crafts acylation step leading to diketone (Scheme 1).

### Experimental and computational studies of 1,3-diarylisoacenofurans

**Figure 5:**
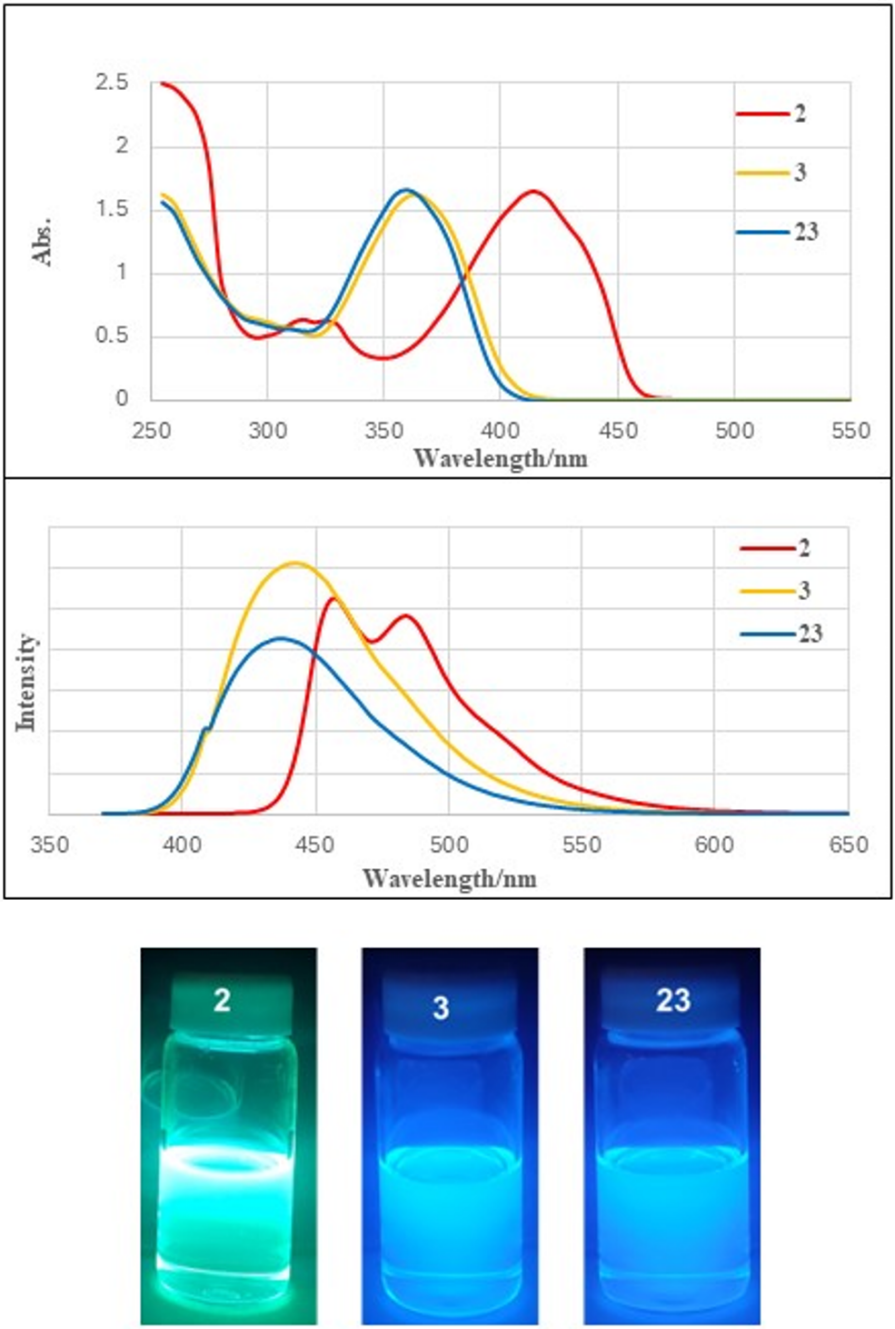
UV–vis (top) and fluorescence (middle) spectra for 10^−6^ M solutions of 1,3-diarylisobenzofurans **2**, **3** and **23** in CH_2_Cl_2_ solvent. The vials of **2**, **3** and **23** showing fluorescence emission (bottom) were excited at 365 nm.

Purified isobenzofurans **2**, **3** and **23** were studied by UV–vis and fluorescence spectroscopies (Figure 5). Compound **2** is devoid of *ortho* groups on its 1,3-diphenyl substituents and shows the longest wavelengths of absorption (λ_max_ = 415 nm) and emission (emission λ_max_ = 484 nm) in this series, consistent with a more highly conjugated π-system in which the 1,3-diphenyl substituents lie flat or nearly flat relative to the isobenzofuran backbone. Likewise, compound **2** is yellow while compounds **3** and **23** are colorless. Compounds **3** and **23** show similar absorption (λ_max_ = 364 and 360 nm for **3** and **23**, respectively) and emission (λ_max_ = 442 and 436 nm for **3** and **23**, respectively) spectra, both consistent with a less conjugated π-system compared to **2**. This is due entirely to out-of-plane rotation of the sterically congesting 1,3-diaryl groups (Figure 4).

**Figure 6:**
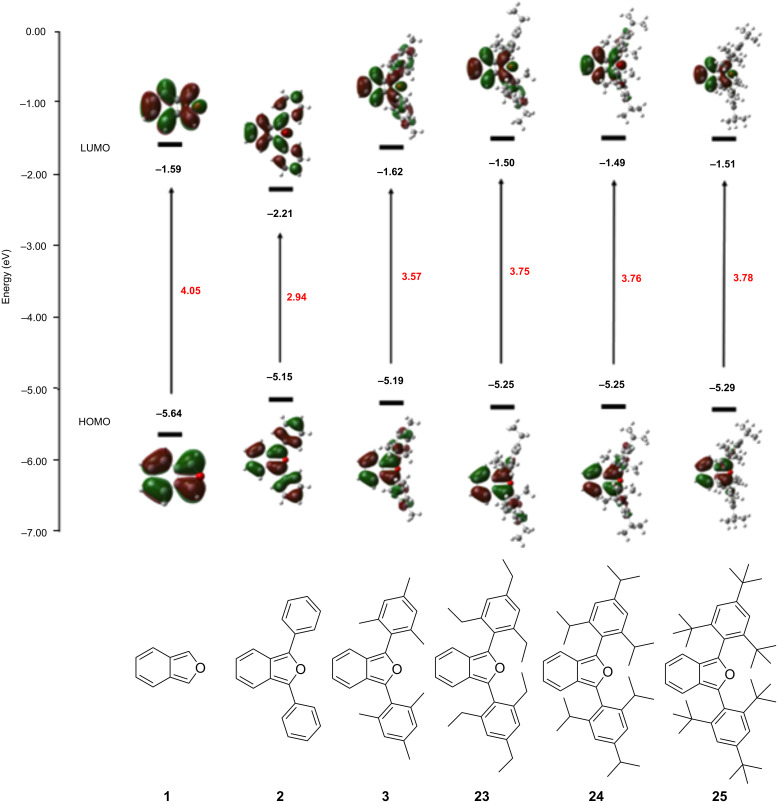
Calculated HOMO and LUMO orbitals for parent isobenzofuran (**1**) and 1,3-diarylisobenzofuran derivatives **2**, **3**, **23**, **24** and **25** using a DFT method performed at the B3YLP/6-311+G(d,p)//B3YLP/6-31G(d) level using Gaussian 09 [12].

In order to further probe the impact of sterically congesting 1,3-diaryl substituents in 1,3-diarylisobenzofurans, we studied compounds **1**, **2**, **3**, **23**, **24** and **25** (Figure 6) using a DFT method. The calculated HOMO and LUMO orbitals are shown in Figure 6. Here, the impacts of 1,3-diaryl substituents are illuminated. Thus, both HOMO and LUMO orbital densities for **2** are spread throughout the molecule’s entire π-system including the 1,3-diphenyl substituents. This extensive delocalization of orbital density significantly raises the HOMO energy level and lowers the LUMO energy level compared to parent isobenzofuran (**1**). Conversely, both the HOMO and LUMO orbitals for compound **3** with 1,3-dimesityl substituents show reduced orbital density on the mesityl substituents compared to the phenyl substituents of **2**. Likewise, **3** possesses a lower energy HOMO orbital and a higher energy LUMO orbital compared to **2**. The π-systems for compounds **23**, **24** and **25** with 2’,4’,6’-triethylphenyl, 2’,4’,6’-triisopropylphenyl and 2’,4’,6’-tri-*tert*-butylphenyl substituents are quite similar to each other. Each shows little or no HOMO or LUMO orbital densities on their respective 1,3-diaryl groups indicating greater out-of-plane rotation compared to **3** and modestly larger HOMO–LUMO gaps.

**Figure 7:**
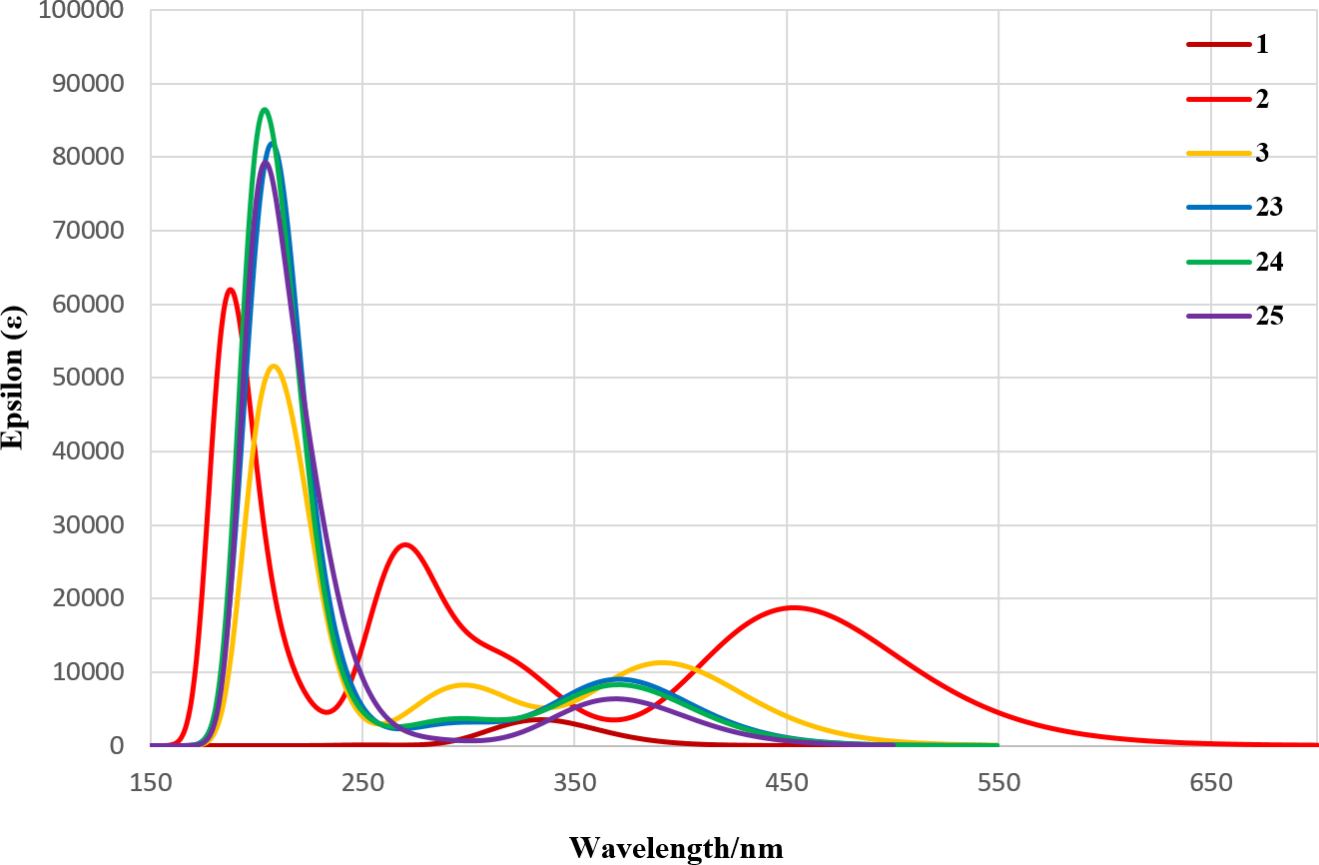
UV–vis spectra calculated for 1,3-diarylisobenzofuran derivatives **1**, **2**, **3**, **23**, **24** and **25** using a DFT method performed at the B3YLP/6-311+G(d,p)//B3YLP/6-31G(d) level using Gaussian 09 [12].

The calculated UV–vis spectra for this series are shown in Figure 7. Compounds **23**, **24** and **25** show nearly identical spectra indicating that each hindered 1,3-diaryl ring is similarly rotated out of plane leading to π-systems with similar HOMO–LUMO gaps. These findings inform any design strategy leading to large, persistent isoacenofurans. Thus, unless there is a need for improved kinetic stabilization of the most reactive 1,3-carbons on the furan ring, there appears little benefit of utilizing highly hindered 2’,4’,6’-triisopropyl (compound **24**) or 2’,4’,6’-tri-*tert*-butyl (compound **25**) substituents, especially as these compounds are considerably more difficult to synthesize compared to **3** and **23**.

### Relative rates for the reactions between 1,3-diarylisobenzofurans **2**, **3** and **23** with the strong dienophiles dimethyl acetelyenedicarboxylate (DMAD) and acrylonitrile

**Figure 8:**
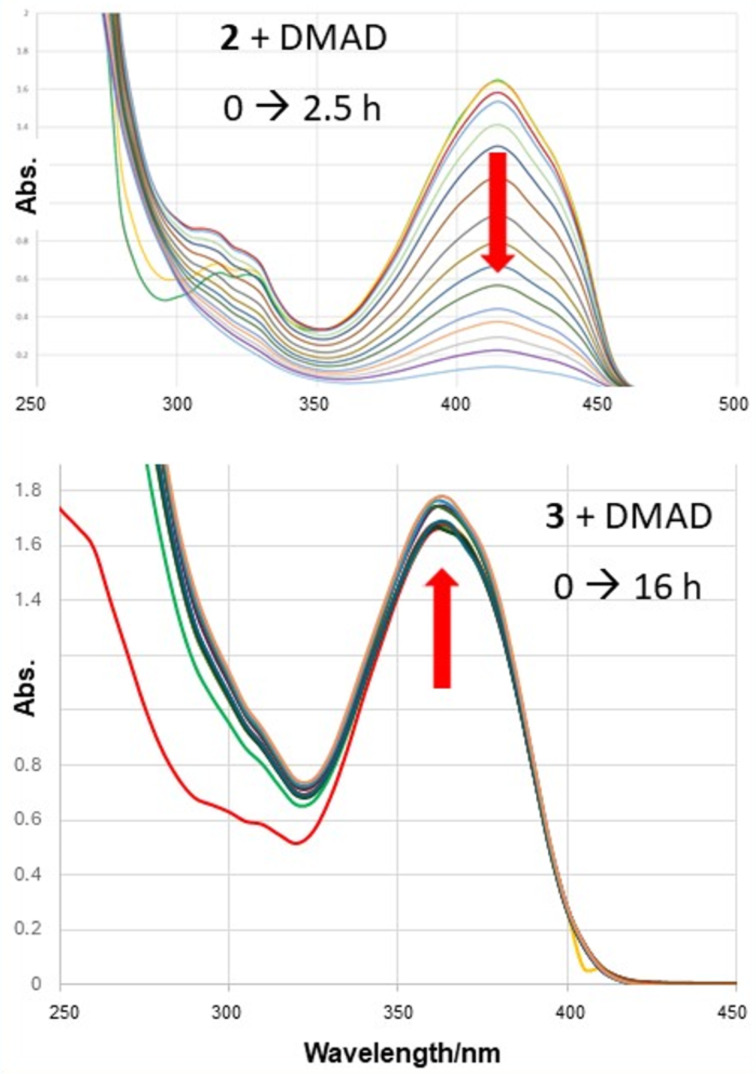
UV–vis spectra for the reactions of **2** (top) and **3** (bottom) with a 7000-fold excess of DMAD in CH_2_Cl_2_ solvent at room temperature.

We studied the reactions of **2**, **3** and **23** under pseudo-1st order kinetic conditions by utilizing a 7000-fold excess of dimethyl acetylenedicarboxylate (DMAD) at room temperature. The reactions were monitored by UV–vis spectroscopy. Compound **2** undergoes rapid reaction with DMAD under these conditions and is more than 90% consumed after 2.5 hours (Figure 8, top). Conversely, compounds **3** (Figure 8, bottom) and **23** are unreactive under these conditions, even after extended periods of time. The absorptions for **3** and **23** actually increased slowly over extended time due to the gradual evaporation of CH_2_Cl_2_ solvent in the capped UV–vis cuvette. Similar results were obtained upon switching the dienophile from DMAD to acrylonitrile. Once again, compounds **3** and **23** were unreactive, even after 184 hours of reaction time with a 13,500-fold excess of acrylonitrile.

The reaction between **3** and a large excess of DMAD (116 equivalents) in boiling toluene (111 °C) was also studied by ^1^H NMR spectroscopy. After 51 hours of reaction in boiling CH_2_Cl_2_, Diels–Alder adduct **27** was observed in 22% yield. Compound **27** was identified by ^1^H NMR and ^13^C NMR spectroscopies as well as high-resolution ESI mass spectrometry.

**Scheme 2:**
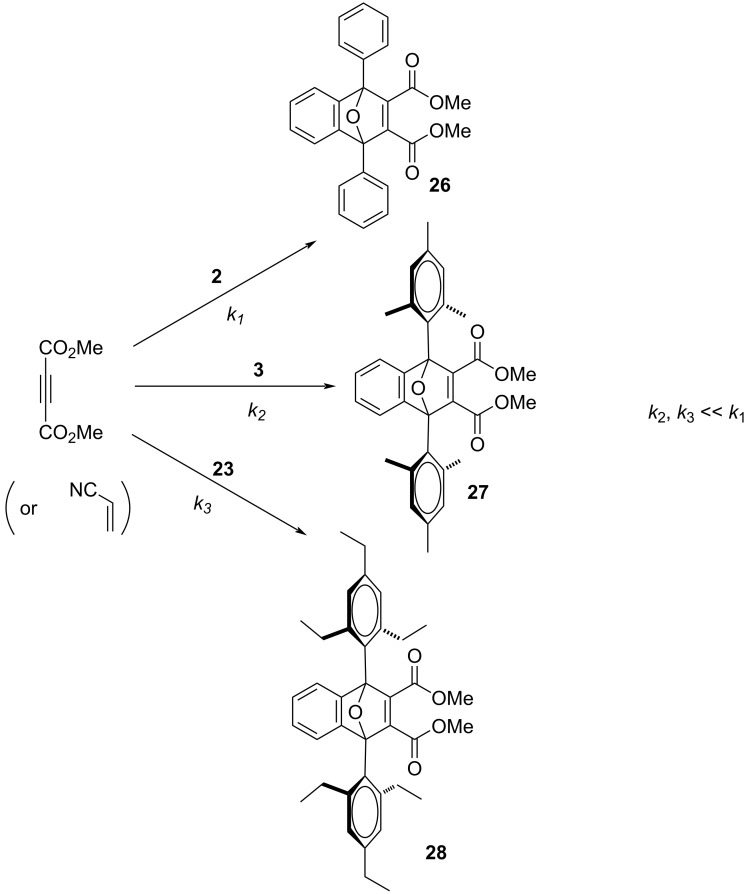
Reactions between 1,3-diarylisobenzofurans **2**, **3** and **23** and DMAD to produce Diels–Alder adducts **26**, **27** and **28**.

Although the lack of reactivity observed for **3** and **23** limited our kinetic analysis, we can nonetheless conclude that the rates of reactions between either **3** or **23** and either of the strong dienophiles, DMAD or acrylonitrile, are at least two orders of magnitude slower than the corresponding reactions involving **2** (Scheme 2). Clearly, the non-planar geometry of the mesityl (Figure 4) and 2’,4’,6’-triethylphenyl substituents in compounds **3** and **23**, respectively, provides for enhanced steric congestion at the most reactive 1,3-carbons of the furan ring. Likewise, **3** and **23** are stable indefinitely in the solid state and persist for days in solution phase without significant decomposition (see Supporting Information File 1). The mesityl and 2’,4’,6’-triethylphenyl substituents both provide a strong measure of kinetic stabilization. Solution phase stability is particularly important if isoacenofurans are to be utilized as thin-film organic semiconductors cast from solution.

## Conclusion

We utilized DFT calculations to demonstrate that isoacenofurans of any size possess smaller HOMO–LUMO gaps than the corresponding acene bearing an isoelectronic π-system. This finding alone provides compelling reasons to attempt the synthesis of large, persistent isoacenofurans for use as organic semiconductors. However, such syntheses must account for the projected lack of stability associated with isoacenofurans. We demonstrated that sterically congesting *ortho* groups on 1,3-diarylisobenzofurans like **3** and **23** force the molecules to adopt non-planar conformations in which the aryl groups rotate out-of-plane. The out-of-plane rotations provide for enhanced steric congestion both above and below the most reactive 1,3-carbons of the furan ring, dramatically reducing their reactivity with dienophiles including DMAD, acrylonitrile and singlet oxygen. Due to these non-planar conformations, the HOMO and LUMO orbitals for **3** and **23** and related compounds show reduced orbital densities on their aryl substituents, effectively reducing π-conjugation and raising HOMO–LUMO gaps. There appears little benefit to utilizing highly hindered 2’,4’,6’-triisopropylphenyl groups as in **24** or 2’,4’,6’-tri-*tert*-butylphenyl groups as in **25**, especially as (i) isoacenofurans with these substituents are considerably more difficult to synthesize, and (ii) compounds **3** and **23** already provide excellent kinetic stabilization to the most reactive 1,3-carbons of the furan ring. Like acenes, large isoacenofurans will be prone to photooxidation along their acene-like backbones, necessitating additional, strategically placed substituents that are known to slow photooxidation. The insights gained here suggest that the synthesis of large, persistent, kinetically stabilized isoacenofurans with unusually small HOMO–LUMO gaps is achievable. As such, these molecules deserve increased attention as a new class of organic semiconductors.

## Experimental

### Materials and methods

Commercial reagents and solvents were purchased from Sigma-Aldrich, Alfa Aesar, TCI America or Thermo Fisher Scientific, and used as received. Dry solvents were obtained using a solvent purification system (Innovative Technologies, Inc.) and handled under a nitrogen atmosphere, unless otherwise noted. Flash chromatography was performed using SiliaFlash^®^ F60 40–63 µm (230–400 mesh) 60 Å silica from Silicycle Inc. and RediSep^®^ Rf Silica Flash Columns (12 g, 24 g or 40 g) on a CombiFlash^®^ Rf 200 instrument (Teledyne Isco, Inc.). Evaporation of solvents was accomplished using an IKA^®^ RV 10 digital rotary evaporator. Baker-flex^®^ silica gel IB2-F thin-layer chromatography (TLC) plates were purchased from J.T. Baker. A 4 watt 254 nm lamp (Analtytik Jena Co.) and a modified cardboard box were utilized for detection of TLC spots. Melting points were determined in open capillary tubes using a Mel-Temp apparatus, and are uncorrected. Proton nuclear magnetic resonance (^1^H NMR) spectra and carbon nuclear magnetic resonance (^13^C NMR) spectra were recorded on either a Bruker 500 MHz or Bruker 700 MHz nuclear magnetic resonance spectrometer using 5 mm NMR tubes with plastic caps. High-resolution mass spectra (HRMS) were obtained on a Thermo Scientific Vanquish UHPLC and Exploris 120 Mass Spectrometer at the University of New Hampshire’s University Instrumentation Center using a peak-matching protocol to determine the mass and error range of the molecular ion, and employing electrospray as the ionization technique. UV–vis absorption spectra were measured with a Varian Cary 50 Scan UV–visible spectrophotometer and corrected for background signal with a solvent-filled cuvette. Fluorescence spectra were measured on a FS5 spectrofluorometer (150 W CW Ozone-free xenon arc lamp) from Edinburgh Instruments.

### 1,3-Dimesitylisobenzofuran (**3**)

**Scheme 3:**
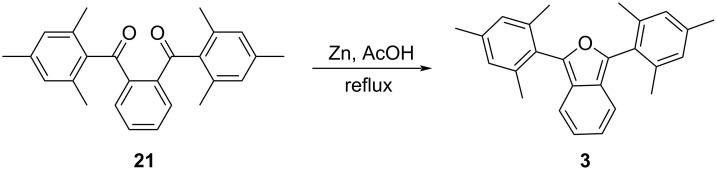
Synthesis of 1,3-dimesitylisobenzofuran (**3**).

To a round bottom flask was added 1,2-phenylenebis(mesitylmethanone) (**21**, 0.10 g, 0.27 mmol), zinc dust (0.70 g, 11 mmol) and 10 mL glacial acetic acid. After attaching a reflux condenser, the mixture was heated to reflux for 12 hours with stirring. The hot reaction solution was filtered. To the hot filtrate was added 5 mL of cold water leading to the precipitation of crude product. The crude product was vacuum filtered, washed with 5 mL water, and then air dried to give **3** as a white solid (57 mg, 60%, Scheme 3). Mp 169–170 °C (from [13], 188–189 °C); ^1^H NMR (500 MHz, CDCl_3_) δ 7.15–7.08 (m, 2H), 6.98 (s, 4H), 6.86–6.79 (m, 2H), 2.35 (s, 6H), 2.12 (s, 12H); ^13^C NMR (126 MHz, CDCl_3_) δ 143.54, 139.17, 138.71, 128.23, 127.43, 123.39, 121.93, 119.78, 21.22, 20.49; UV–vis λ_max_ (7 × 10^−5^ M in CH_2_Cl_2_): 364.3 nm; HRESIMS: calcd for [M + H]^+^, 355.2062; found, 355.2042. For an alternative synthesis, see [13].

### 1,2-Phenylenebis(mesitylmethanone) (**21**)

**Scheme 4:**
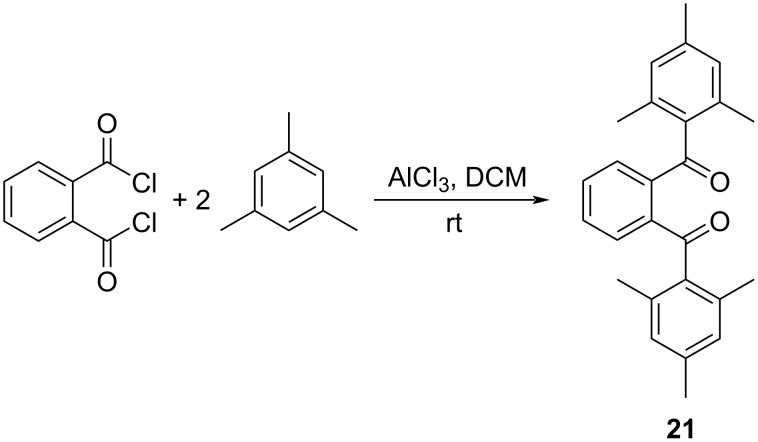
Synthesis of 1,2-phenylenebis(mesitylmethanone) (**21**).

To a 100 mL round bottom flask equipped with a stir bar was added anhydrous aluminum chloride (0.26 g, 2.0 mmol) and 10 mL of CH_2_Cl_2_ solvent. An addition funnel was attached and to this was added phthaloyl dichloride (0.20 g, 0.99 mmol) and 5 mL CH_2_Cl_2_. The content of the addition funnel was added dropwise into the round bottom flask over 5 minutes with stirring. The addition funnel was reloaded with mesitylene (0.215 g, 1.79 mmol) and an additional 5 mL CH_2_Cl_2_. The content of the addition funnel was once again added dropwise into the round bottom flask over 5 minutes with stirring. The light-yellow solution turned to dark brown. After 15 min, 10 mL of a saturated aqueous solution of NaCl was added to quench the reaction. The content of the flask was transferred to a 125 mL separatory funnel and extracted twice with 20 mL of CH_2_Cl_2_. The organic extracts were combined, dried over anhydrous Na_2_SO_4_ and gravity filtered. The solvent was evaporated at reduced pressure leaving a yellow solid as crude product. The crude product was recrystallized using 10 mL of hexane to obtain **21** as a crystalline white solid (0.33 g, 99%, Scheme 4). Mp 234–235 °C (from [14], 237–238 °C); ^1^H NMR (500 MHz, CDCl_3_) δ 7.48–7.36 (m, 4H), 6.86 (s, 4H), 2.30 (s, 6H), 2.19 (s, 12H); ^13^C NMR (126 MHz, CDCl_3_) δ 199.60, 141.23, 139.54, 136.54, 136.07, 131.22, 130.38, 129.00, 21.17, 20.29. For an alternative synthesis, see [14].

### 1,2-Phenylenebis((2,4,6-triethylphenyl)methanone) (**22**)

**Scheme 5:**
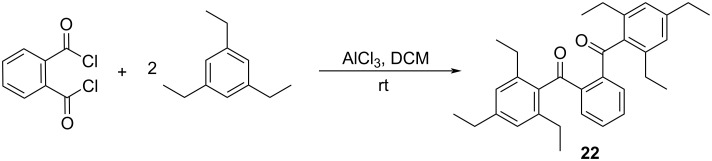
Synthesis of 1,2-phenylenebis((2,4,6-triethylphenyl)methanone) (**22**).

To a 100 mL round bottom flask equipped with a stir bar was added anhydrous aluminum chloride (0.66 g, 4.9 mmol) and 10 mL of CH_2_Cl_2_ solvent. An addition funnel was attached and to this was added phthaloyl dichloride (0.56 g, 2.8 mmol) and 5 mL CH_2_Cl_2_. The content of the addition funnel was added dropwise into the round bottom flask over 5 minutes with stirring. The addition funnel was reloaded with 1,3,5-triethylbenzene (0.90 g, 5.5 mmol) and an additional 5 mL CH_2_Cl_2_. The content of the addition funnel was once again added dropwise into the round bottom flask over 5 minutes with stirring. The light-yellow solution turned to dark brown. After 30 min, 10 mL of a saturated aqueous solution of NaCl was added to quench the reaction. The contents of the flask were transferred to a 125 mL separatory funnel and extracted twice with 20 mL of CH_2_Cl_2_. The organic extracts were combined, dried over anhydrous Na_2_SO_4_ and gravity filtered. The solvent was evaporated at reduced pressure leaving a yellow oil. The oil was purified by silica gel CombiFlash chromatography (hexane/EtOAc 9:1) to obtain **22** as a yellow solid (0.44 g, 35%, Scheme 5). Mp 59–60 °C; ^1^H NMR (500 MHz, CDCl_3_) δ 7.43 (s, 4H), 6.93 (s, 4H), 2.64 (q, *J* = 7.6 Hz, 4H), 2.53 (q, *J* = 7.5 Hz, 8H), 1.25 (t, *J* = 7.6 Hz, 6H), 1.08 (t, *J* = 7.5 Hz, 12H); ^13^C NMR (126 MHz, CDCl_3_) δ 199.43, 145.73, 142.40, 141.03, 135.73, 131.19, 131.05, 125.93, 28.78, 26.50, 15.81, 15.34; HRESIMS: calcd for [M + H]^+^, 455.2950; found, 455.2938.

### 1,3-Bis(2,4,6-triethylphenyl)isobenzofuran (**23**)

**Scheme 6:**
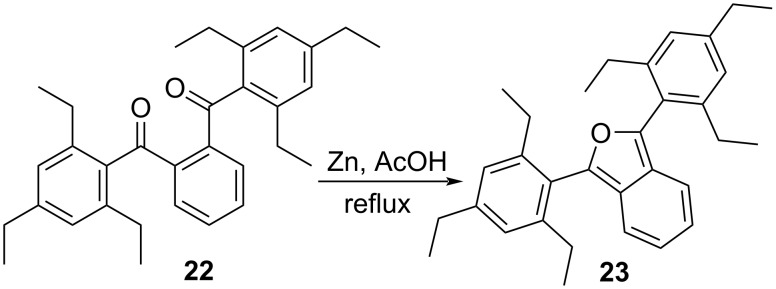
Synthesis of 1,3-bis(2,4,6-triethylphenyl)isobenzofuran (**23**).

To a round bottom flask was added 1,2-phenylenebis((2,4,6-triethylphenyl)methanone) (**22**, 0.20 g, 0.44 mmol), zinc dust (1.15 g, 17.6 mmol) and 13 mL glacial acetic acid. After attaching a reflux condenser, the mixture was heated to reflux for 12 hours with stirring. The hot reaction solution was filtered. To the hot filtrate was added 5 mL of cold water leading to precipitation of crude product. The crude product was vacuum filtered, washed with 5 mL water, and then air dried to give **23** as a white solid (0.12 g, 62%, Scheme 6). Mp 83–84 °C; ^1^H NMR (500 MHz, CDCl_3_) δ 7.15–7.08 (m, 2H), 7.02 (s, 4H), 6.84–6.77 (m, 2H), 2.69 (q, *J* = 7.6 Hz, 4H), 2.44 (m, 8H), 1.30 (t, *J* = 7.6 Hz, 6H), 1.01 (t, *J* = 7.5 Hz, 12H); ^13^C NMR (126 MHz, CDCl_3_) δ 145.90, 145.48, 143.28, 126.29, 125.50, 123.32, 122.18, 119.64, 28.85, 27.14, 15.82, 15.37; UV–vis λ_max_ (7 × 10^−5^ M in CH_2_Cl_2_): 360.2 nm; HRESIMS: calcd for [M + H]^+^, 439.3001; found, 439.2987.

### Dimethyl 1,4-diphenyl-1,4-dihydro-1,4-epoxynaphthalene-2,3-dicarboxylate (**26**)

**Scheme 7:**
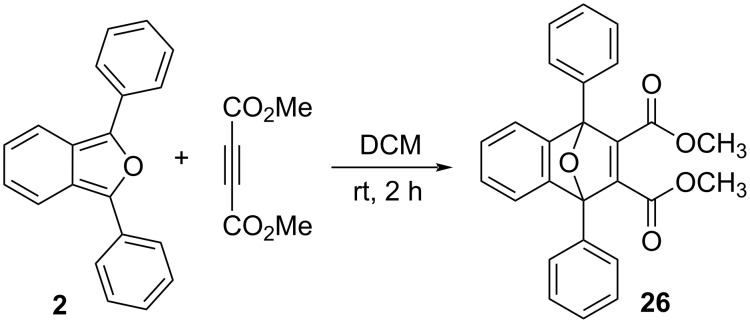
Synthesis of dimethyl 1,4-diphenyl-1,4-dihydro-1,4-epoxynaphthalene-2,3-dicarboxylate (**26**).

In a similar manner to [15], 1,3-diphenylisobenozfuran (**2**, 0.10 g, 0.37 mmol), 5 mL CH_2_Cl_2_ and dimethyl acetylenedicarboxylate (DMAD, 0.116 g, 0.814 mmol) were added to a round bottom flask. The reaction mixture was stirred at room temperature for 2 hours. The solvent was removed by rotary evaporation at reduced pressure to give a light-yellow solid as crude product. The crude product was recrystallized using 5 mL of a hexane/ethanol mixture (10:1) and then air dried to give **26** as a white solid (0.11 g, 72%, Scheme 7). Mp 153.5–154.0 °C (from [15], 181–183 °C); ^1^H NMR (500 MHz, CDCl_3_) δ 7.76–7.70 (m, 4H), 7.53 (m, 2H), 7.50–7.40 (m, 6H), 7.15 (m, 2H), 3.68 (s, 6H); ^13^C NMR (126 MHz, CDCl_3_) δ 164.14, 153.90, 149.13, 133.16, 129.08, 128.58, 128.00, 125.99, 122.20, 94.05, 52.29; UV–vis λ_max_ (1 × 10^−4^ M in CH_2_Cl_2_): 230 nm; HRESIMS: calcd for [M − OCH_3_] ^+^, 381.1127; found, 381.1115.

### Dimethyl 1,4-dimesityl-1,4-dihydro-1,4-epoxynaphthalene-2,3-dicarboxylate (**27**)

**Scheme 8:**
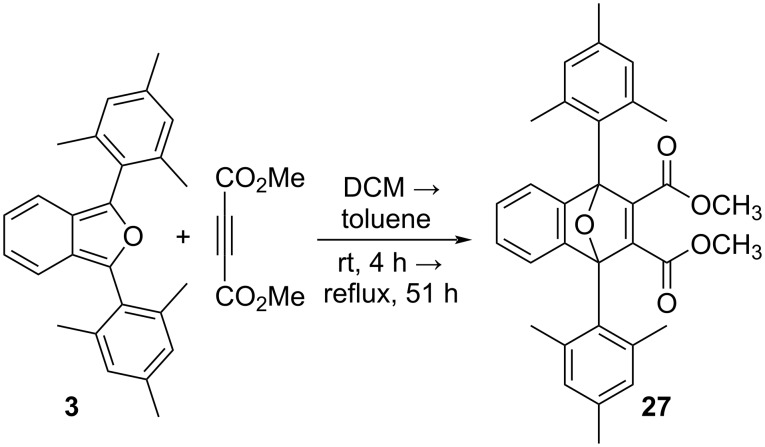
Synthesis of dimethyl 1,4-dimesityl-1,4-dihydro-1,4-epoxynaphthalene-2,3-dicarboxylate (**27**).

To a round bottom flask was added 1,3-dimesitylisobenozfuran (**3**, 0.05 g, 0.14 mmol), 5 mL CH_2_Cl_2_ and dimethyl acetylenedicarboxylate (DMAD, 2.31 g, 16.3 mmol). The reaction mixture was stirred at room temperature for 4 hours. The solvent was removed by rotary evaporation at reduced pressure to give a light-yellow solid. TLC (hexane/EtOAc 3:1) indicated no reaction. Additional DMAD was added to the unreacted mixture in the round bottom flask (2.31 g, 16.3 mmol) along with 2 mL toluene. After attaching a reflux condenser, the mixture was heated to reflux for 51 hours. The mixture was cooled to room temperature and toluene was removed by rotary evaporation at reduced pressure to give a sticky, dark brown solid. The solid was pre-purified by silica gel CombiFlash chromatography (hexane/EtOAc 9:1) to obtain a yellow oil as crude product (23 mg). Finally, the crude product was purified by preparative TLC (petroleum ether/EtOAc 3:1) to give **27** as a yellow powdery solid (15 mg, 22%, 35% based on reacted **3**, Scheme 8). Mp 180 °C (dec.); ^1^H NMR (700 MHz, CDCl_3_) δ 7.29 (m, 2H), 6.98 (m, 2H), 6.88 (s, 4H), 3.64 (s, 6H), 2.30 (s, 12H), 2.28 (s, 6H); ^13^C NMR (176 MHz, CDCl_3_) δ 164.38, 155.13, 151.10, 139.03, 137.93, 130.68, 128.02, 124.72, 124.49, 95.54, 51.98, 23.71, 20.75; HRESIMS: calcd for [M + H]^+^, 497.2328; found, 497.2316.

## Supporting Information

File 1 ^1^H NMR stability studies for compounds **3** and **23**, ^1^H and ^13^C NMR spectra for key compounds and ESI high-resolution mass spectra.

## Data Availability

All data that supports the findings of this study is available in the published article and/or the supporting information to this article.
