# Supplementary material for: Kinetically stabilized 1,3-diarylisobenzofurans and the possibility of preparing large, persistent isoacenofurans with unusually small HOMO–LUMO gaps
Source: Beilstein J Org Chem. 2024 May 17;20:1099–110. doi: 10.3762/bjoc.20.97 (PMC11106668; doi:10.3762/bjoc.20.97)
Supplement: File 1 — 1H NMR stability studies for compounds 3 and 23, 1H and 13C NMR spectra for key compounds and ESI high-resolution mass spectra. [file Beilstein_J_Org_Chem-20-1099-s001.pdf]

BEILSTEIN JOURNAL OF ORGANIC CHEMISTRY

Supporting Information

for

Kinetically stabilized 1,3-diarylisobenzofurans and the possibility of preparing large, persistent isoacenofurans with unusually small HOMO–LUMO gaps

Qian Liu and Glen P. Miller

Beilstein J. Org. Chem. **2024,** *20,* 1099–1110. doi:10.3762/bjoc.20.97

^{1}H NMR stability studies for compounds 3 and 23, ^{1}H and ^{13}C NMR spectra for key compounds and ESI high-resolution mass spectra

License and Terms: This is a supporting information file under the terms of the Creative Commons Attribution License (https://creativecommons.org/licenses/by/4.0). Please note that the reuse, redistribution and reproduction in particular requires that the author(s) and source are credited and that individual graphics may be subject to special legal provisions.
The license is subject to the *Beilstein Journal of Organic Chemistry* terms and conditions: (https://www.beilstein-journals.org/bjoc/terms)

Table of Contents

^{1}H NMR stability study for compounds **3** and **23** ………………………………………………………………………………. S3

^{1}H and ^{13}C NMR spectra of key compounds ……………………………………………………………………………………. S8

Electrospray ionization high-resolution mass spectra of new compounds ……………………………………. S20

Stability study for compounds **3** and **23** maintained in the solid state

When not in use, compounds **3** and **23** were stored in vials that were capped and wrapped in foil. The samples were otherwise not protected from ambient oxygen. Periodically, samples for ^{1}H NMR spectroscopy were prepared.

^{1}H NMR samples were prepared by separately dissolving compounds **3** and **23** in $CDCl_3$. The resulting solutions were placed in standard 5 mm NMR tubes and capped with standard plastic NMR tube caps.

Select spectra are displayed on the following 2 pages.

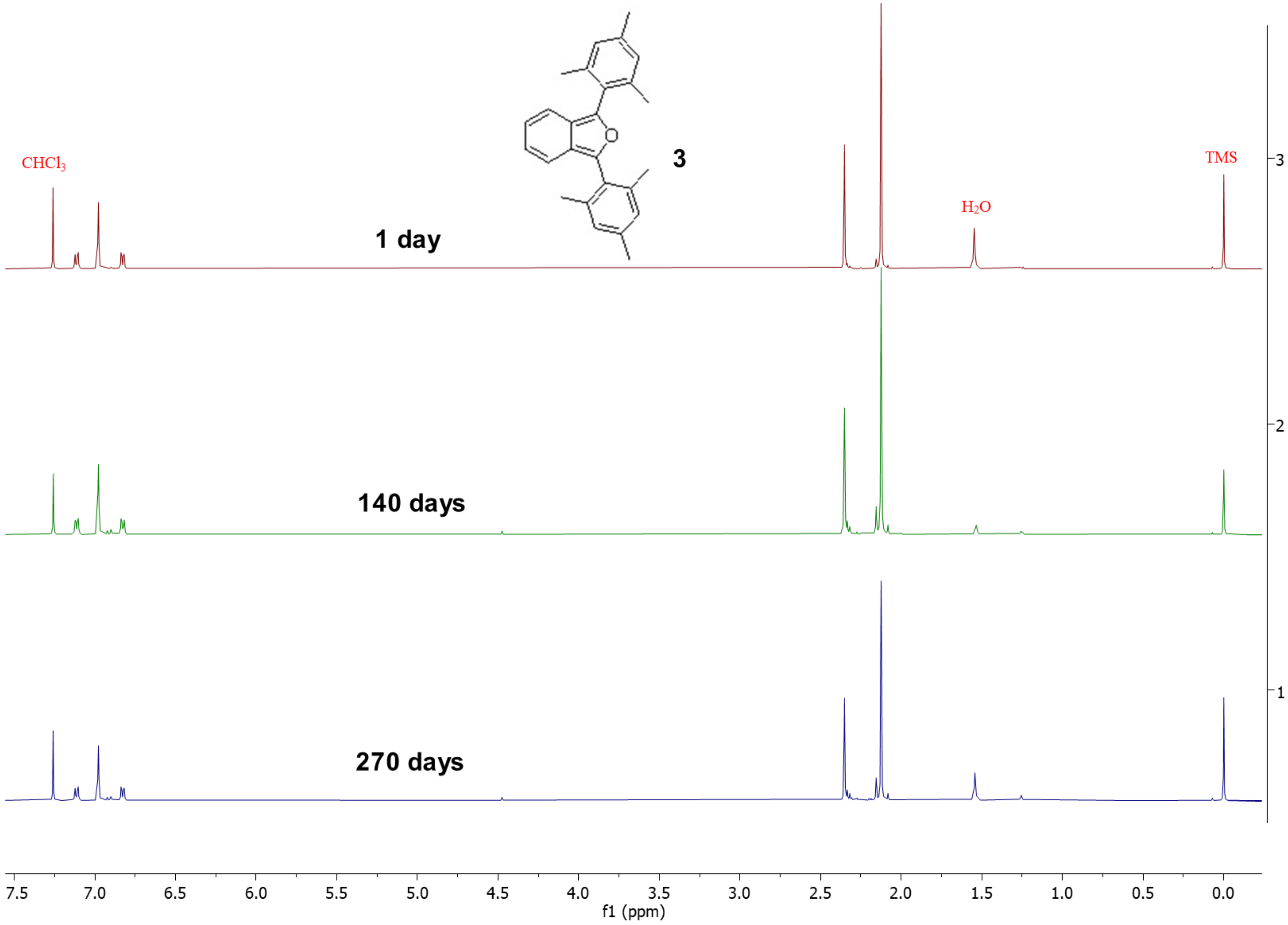

3
CHCl3
1 day
H2O
TMS
140 days
270 days
7.5
7.0
6.5
6.0
5.5
5.0
4.5
4.0
3.5
3.0
2.5
2.0
1.5
1.0
0.5
0.0
f1 (ppm)
3
2
1

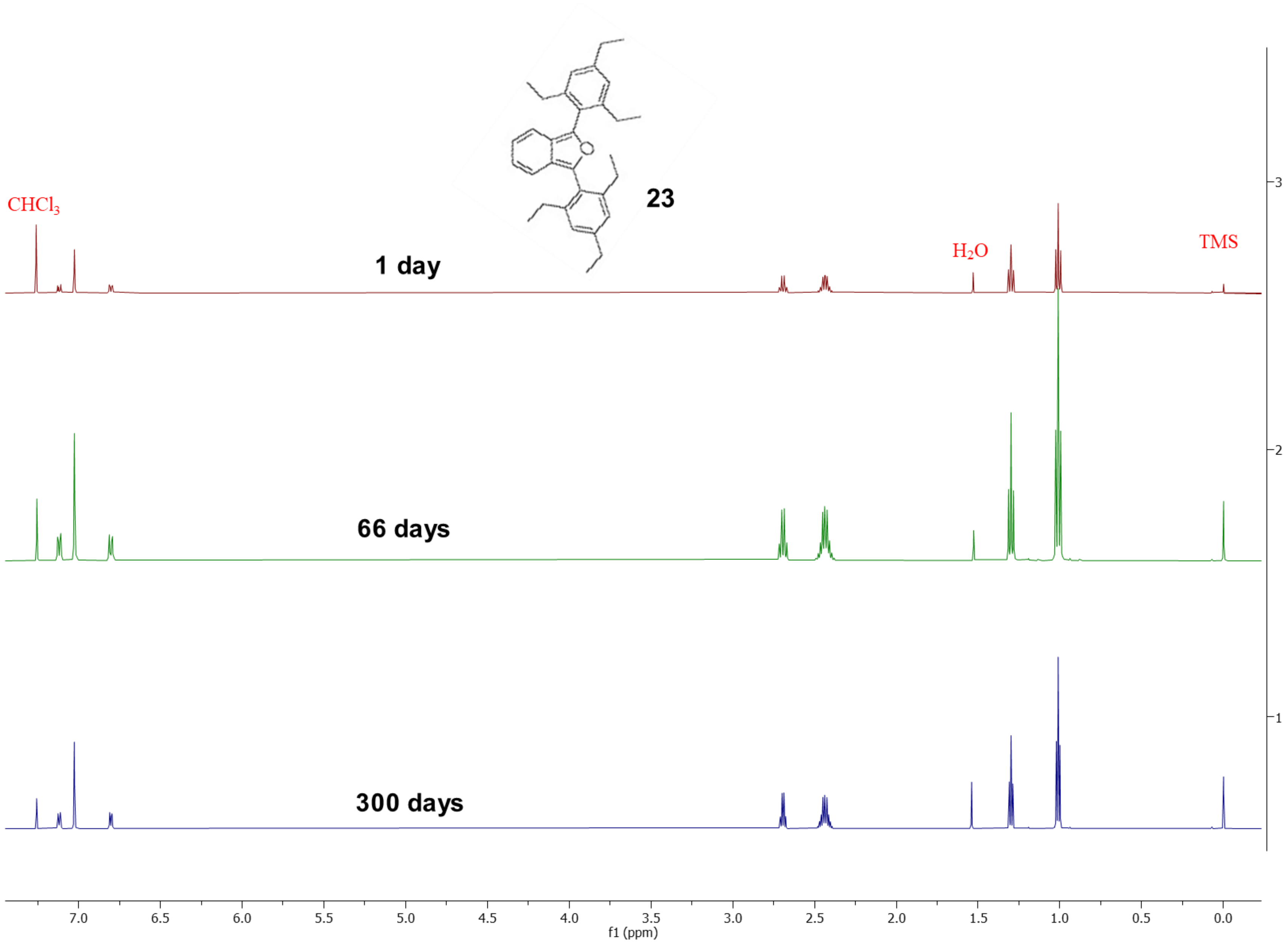

23
CHCl$_3$
H$_2$O
TMS
1 day
66 days
300 days
f1 (ppm)

Stability study for compound **3** maintained in solution

A solution composed of 15 mg of compound **3** was dissolved in 0.6 ml of $CDCl_3$ and placed in a 5 mm NMR tube with plastic cap. The tube was capped but otherwise not protected from air or ambient light. ^{1}H NMR spectra were acquired periodically over 50 days.

Solution Phase Stability of **3**

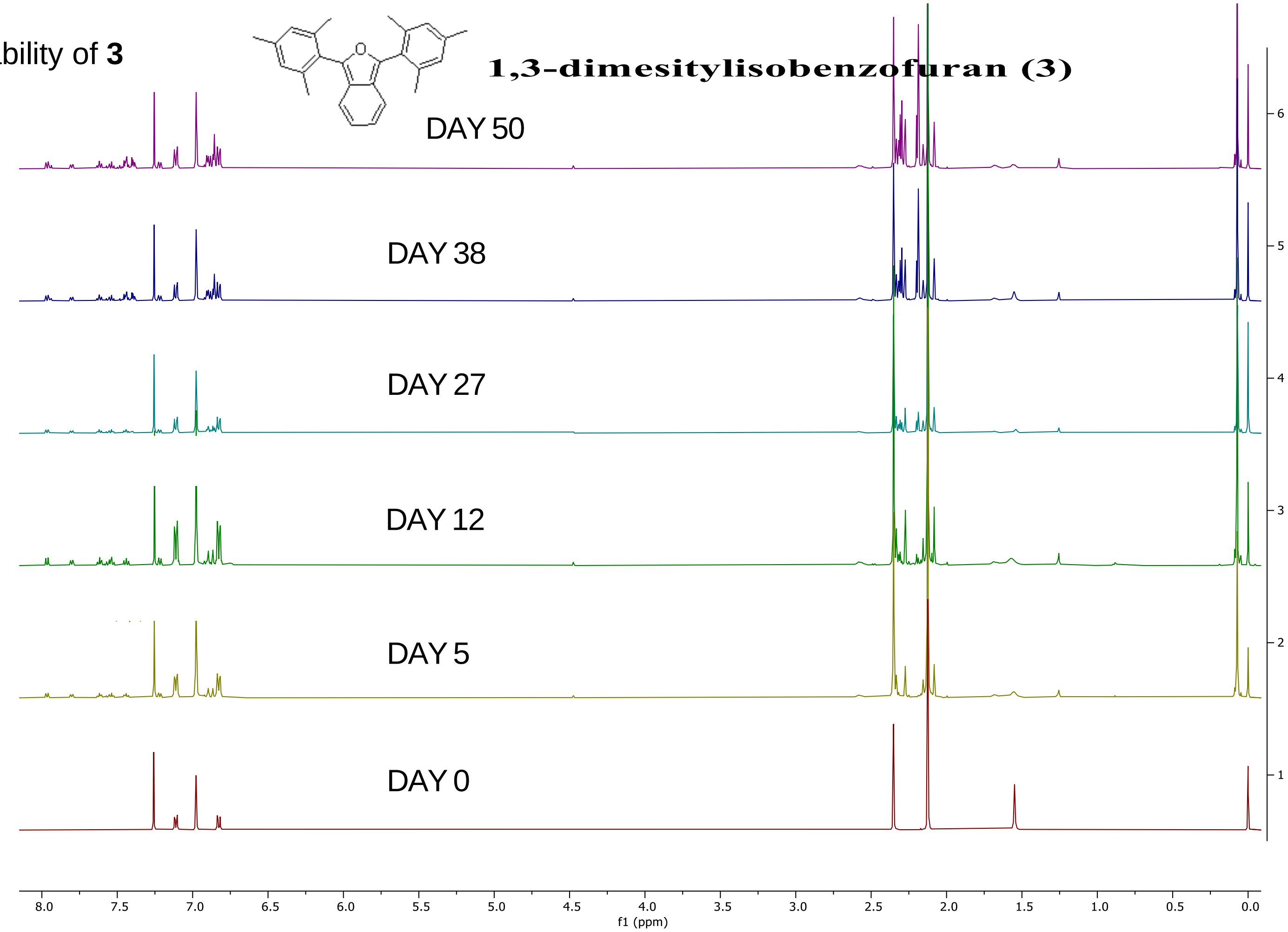

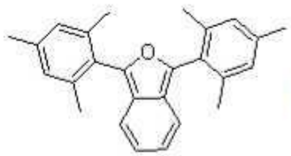

1,3-dimesitylisobenzofuran (3)

^{1}H NMR (500 MHz, $CDCl_3$)

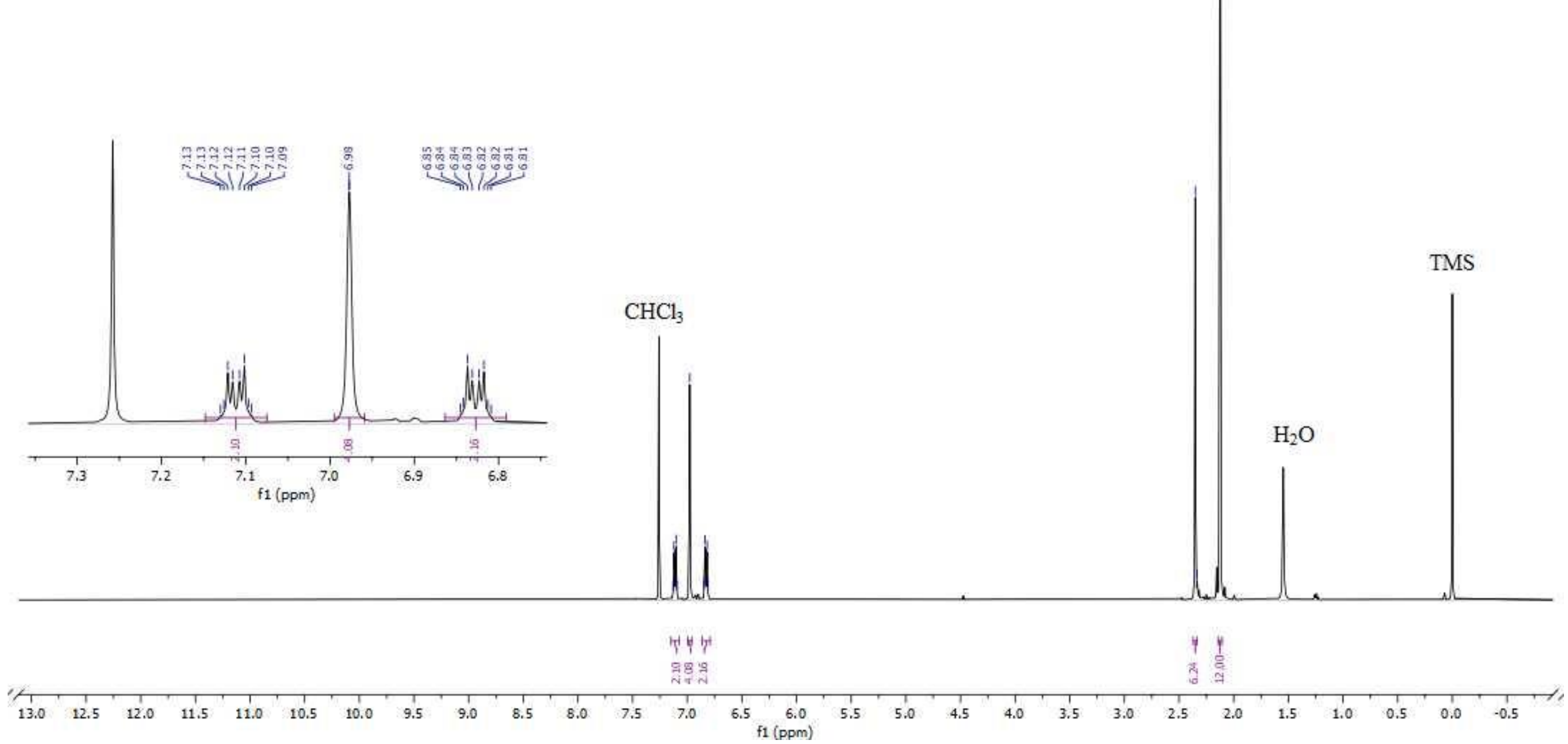

1,3-dimesitylisobenzofuran (3)

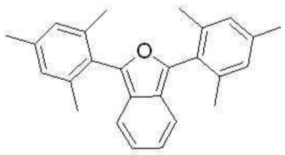

^{13}C NMR (126 MHz, $CDCl_3$) δ 143.54, 139.17, 138.71, 128.23, 127.43, 123.39, 121.93, 119.78, 21.22, 20.49.

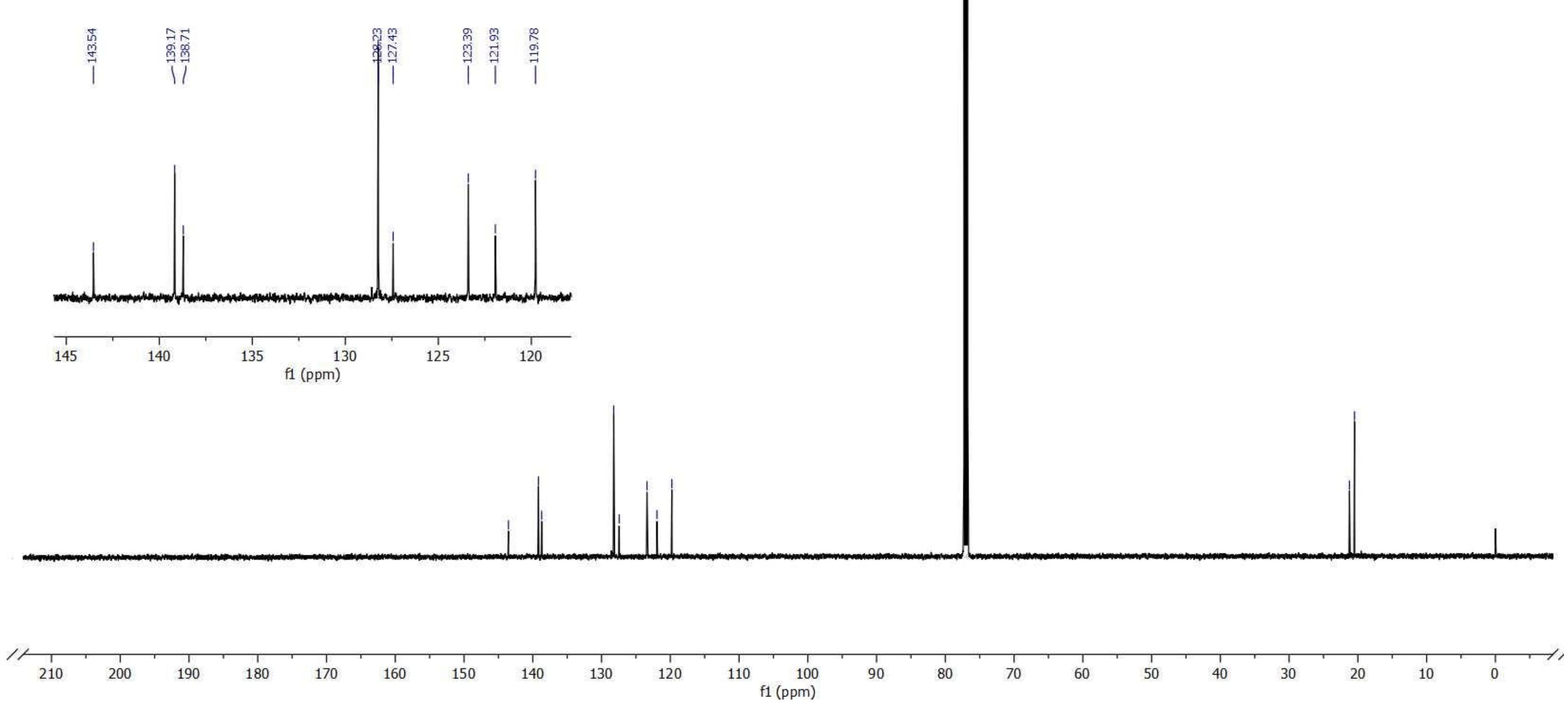

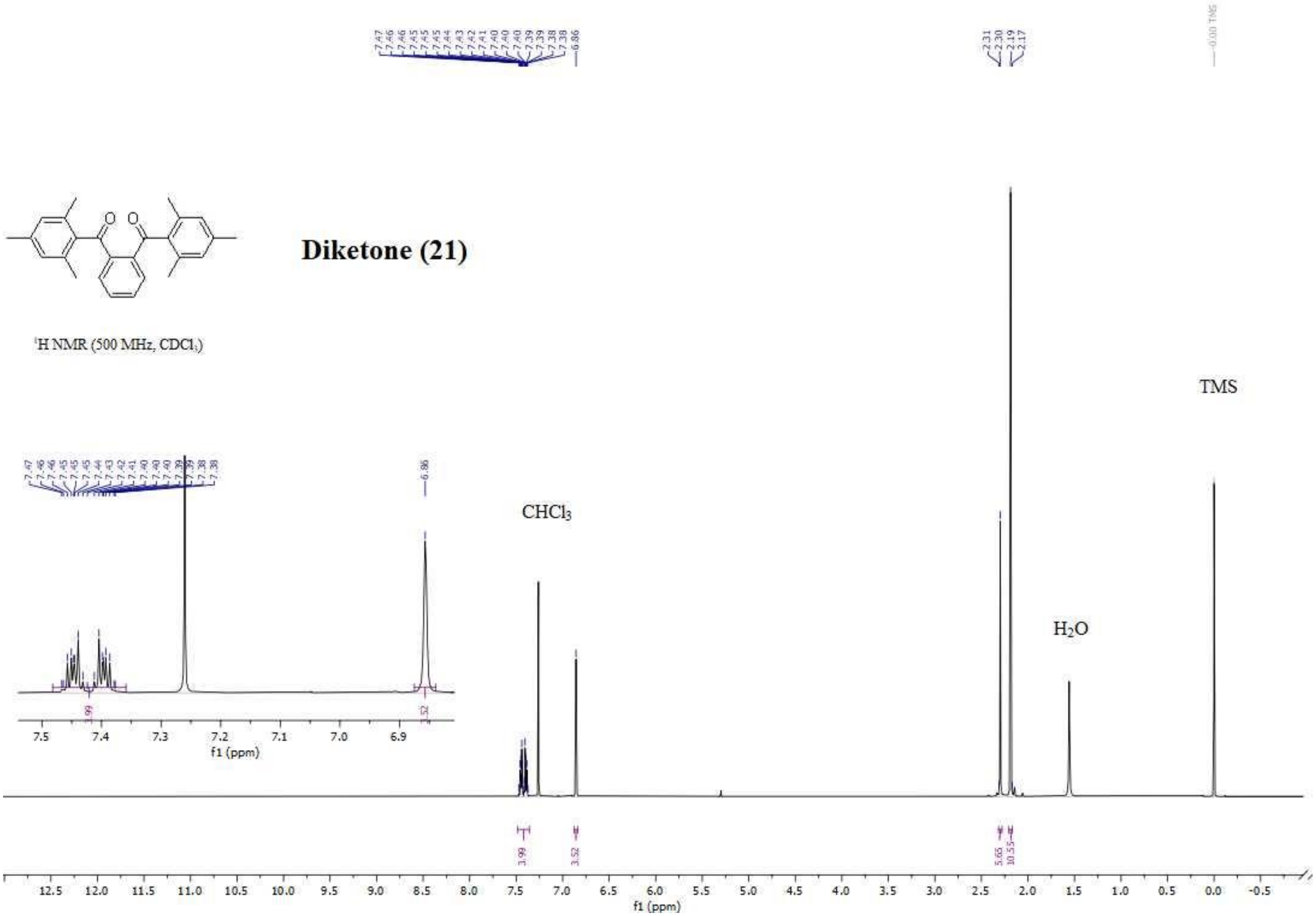
Diketone (21)
¹H NMR (500 MHz, CDCl₃)
CHCl3
H2O
TMS
f1 (ppm)

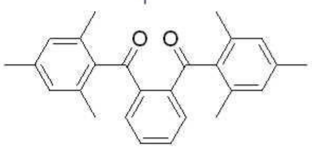

Diketone (21)

^{13}C NMR (126 MHz, $CDCl_3$) δ 199.60, 141.23, 139.54, 136.54, 136.07, 131.22, 130.38, 129.00, 21.17, 20.29.

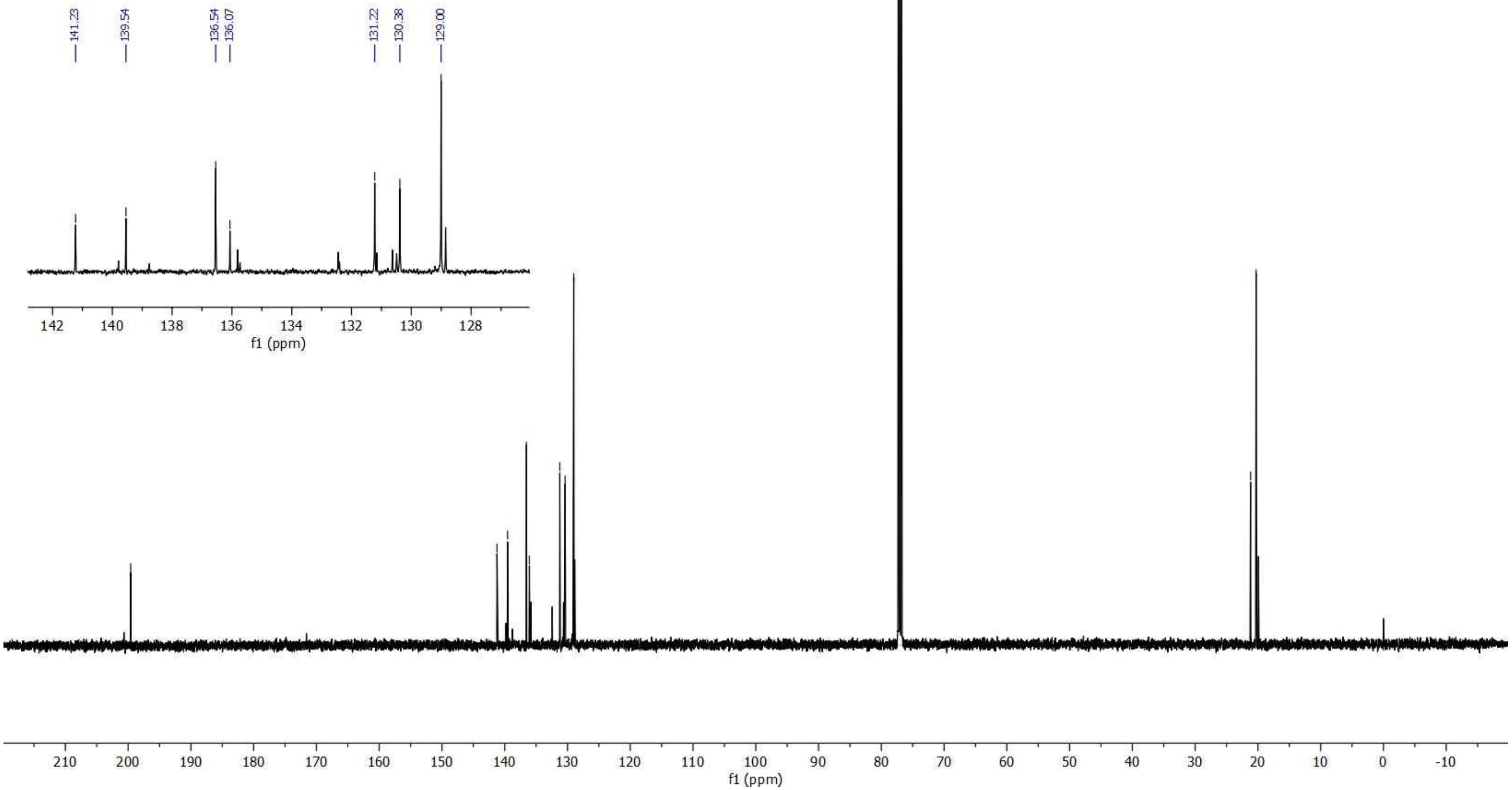

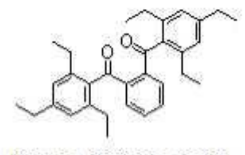

Diketone (22)

^{1}H NMR (500 MHz, $CDCl_3$)

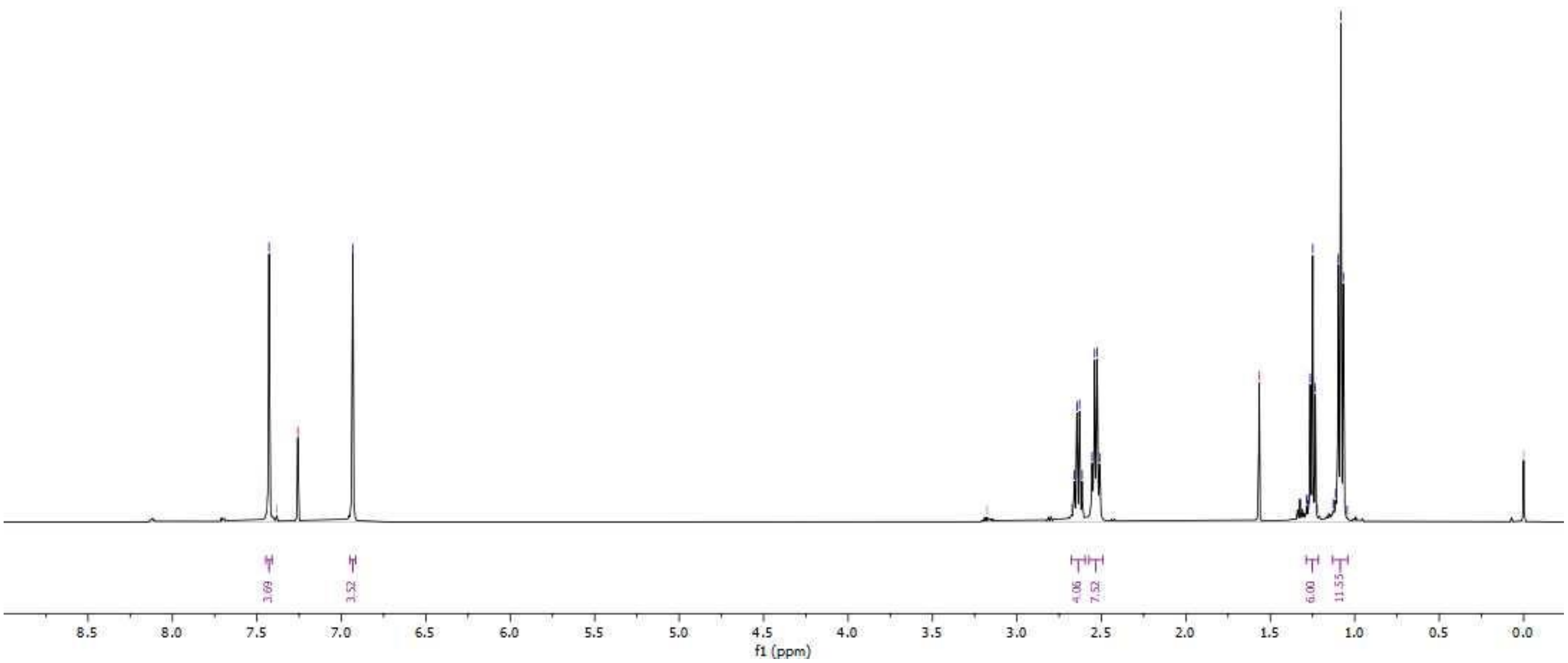

Diketone (22)

^{13}C NMR (126 MHz, $CDCl_3$) δ 199.43, 145.73, 142.40, 141.03, 135.73, 131.19, 131.05, 125.93, 28.78, 26.50, 15.81, 15.34.

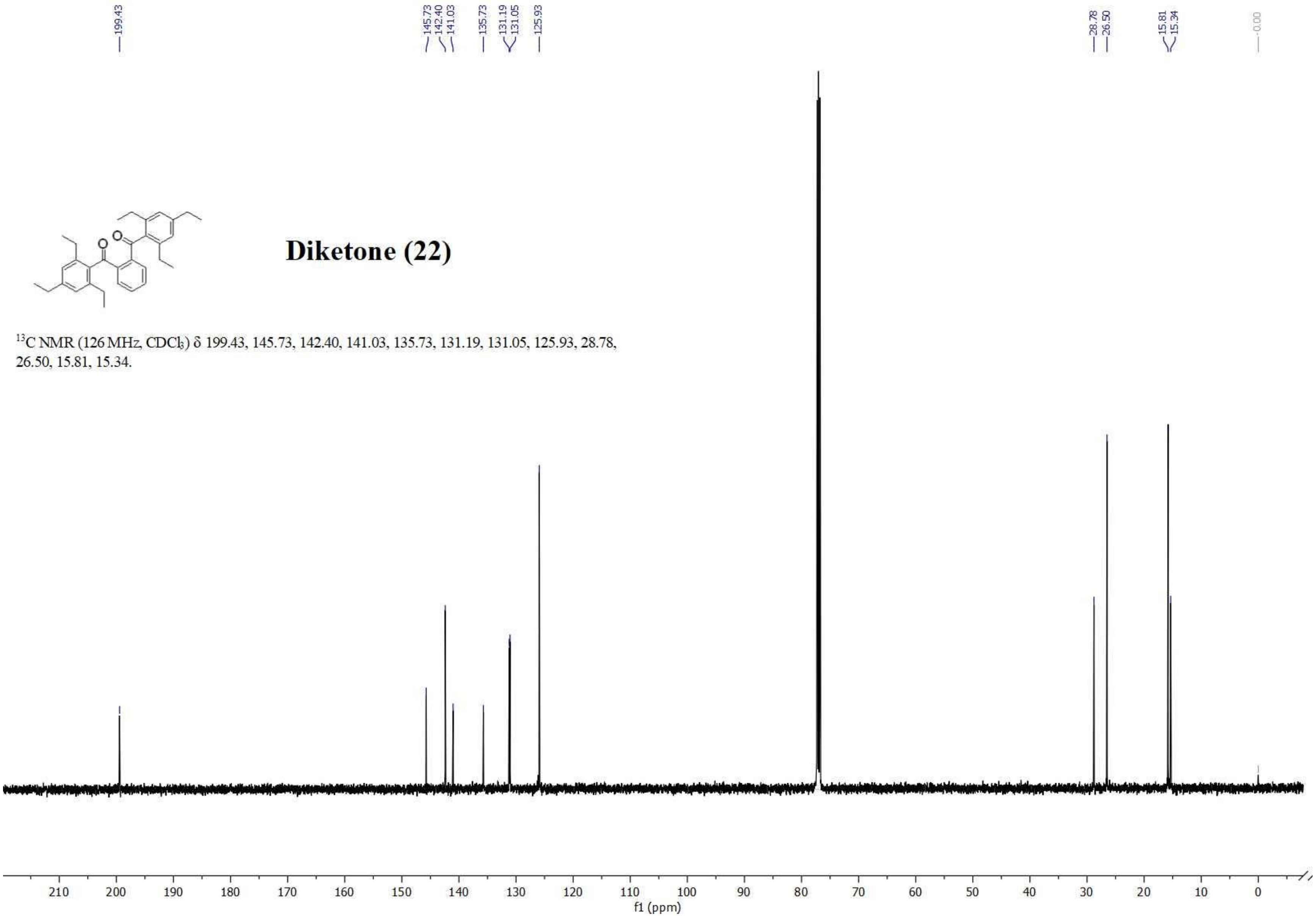

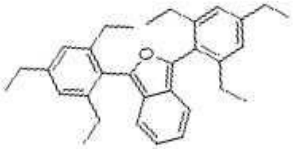

1,3-bis(2,4,6-triethylphenyl)isobenzofuran (23)

^{1}H NMR (500 MHz, $CDCl_3$)

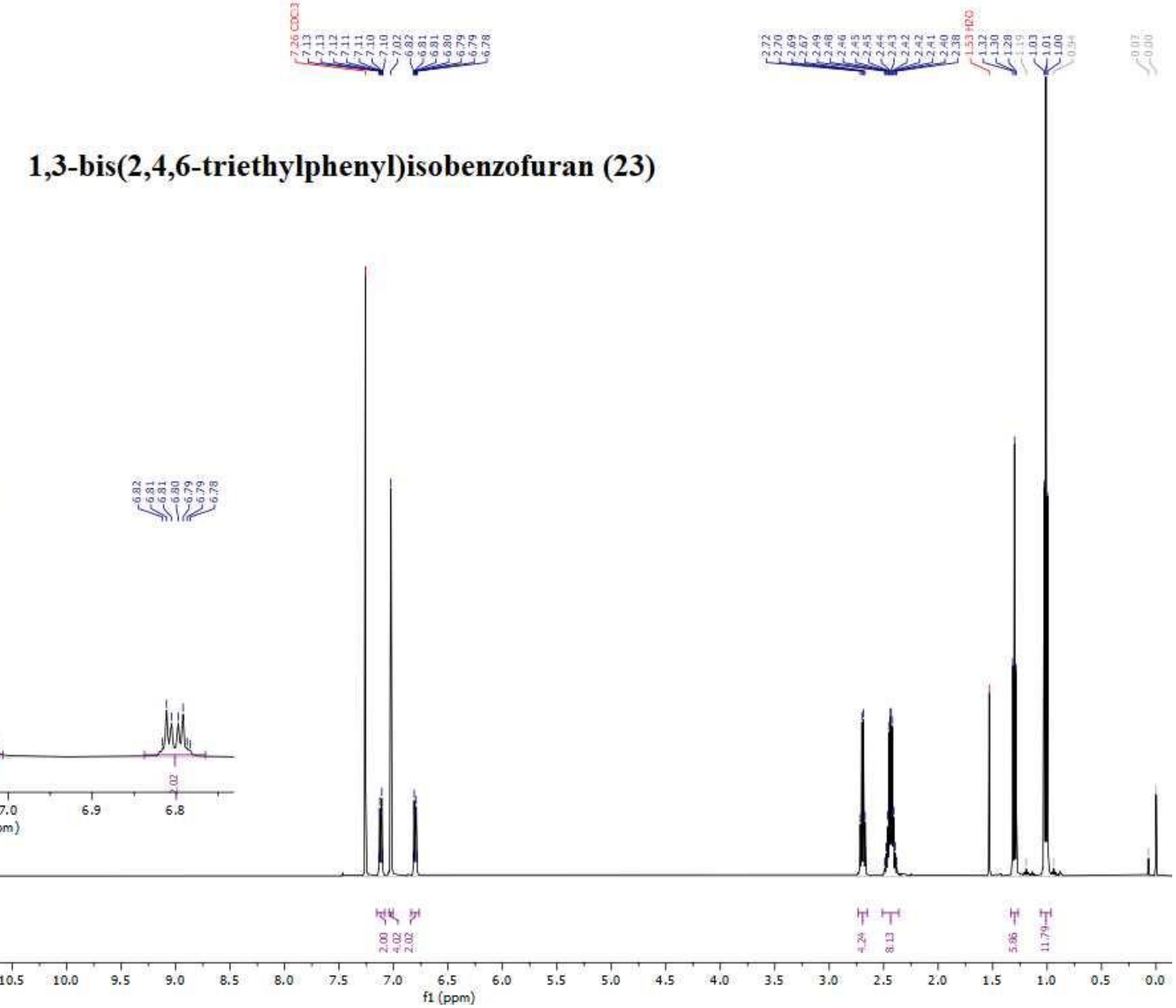

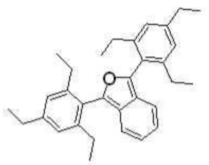

1,3-bis(2,4,6-triethylphenyl)isobenzofuran (23)

^{13}C NMR (126 MHz, $CDCl_3$) δ 145.90, 145.48, 143.28, 126.29, 125.50, 123.32, 122.18, 119.64, 28.85, 27.14, 15.82, 15.37.

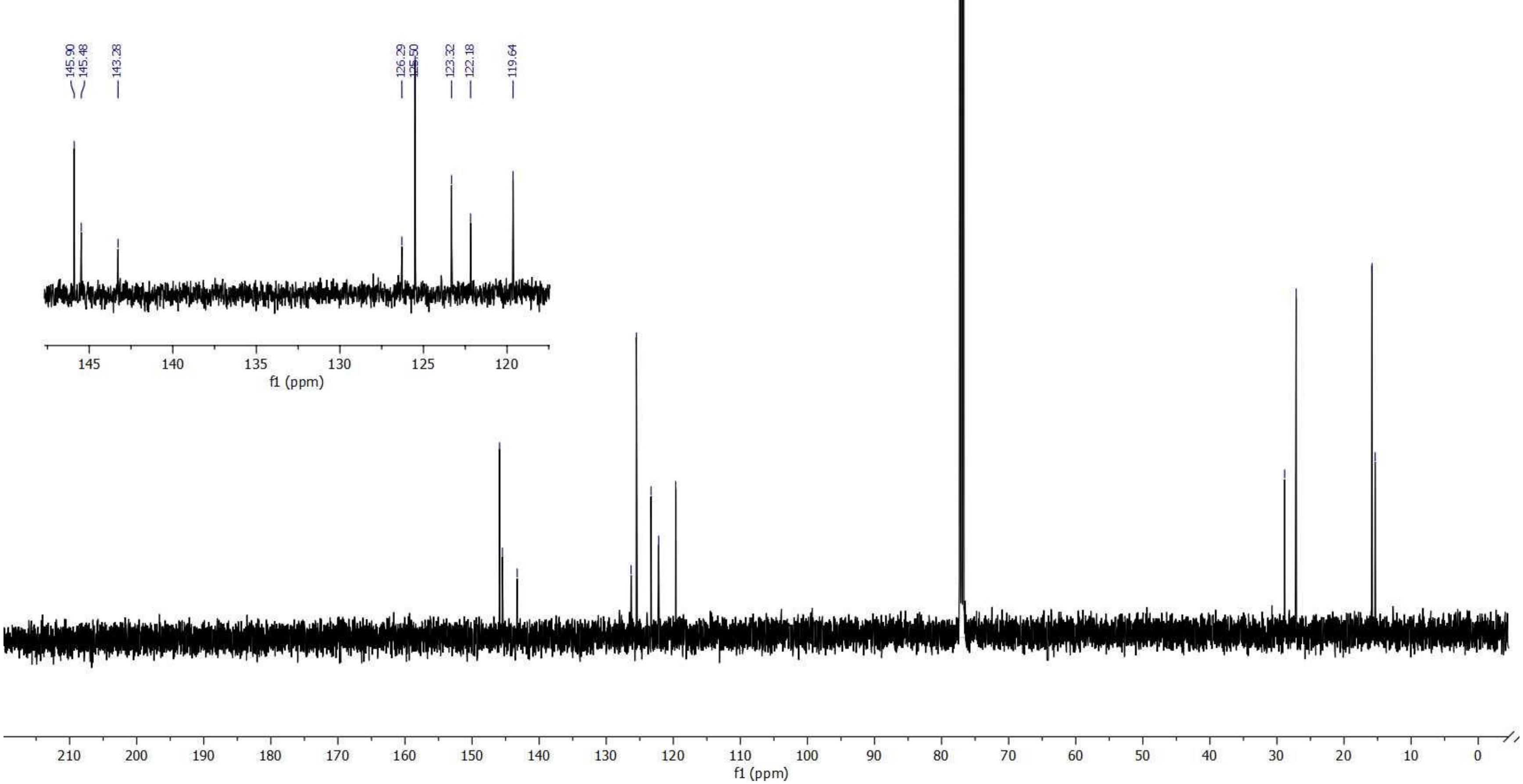

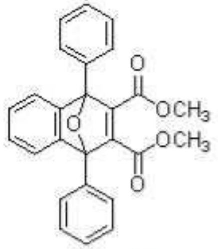

Diels-Alder adduct (26)

^{1}H NMR (500 MHz, $CDCl_3$)

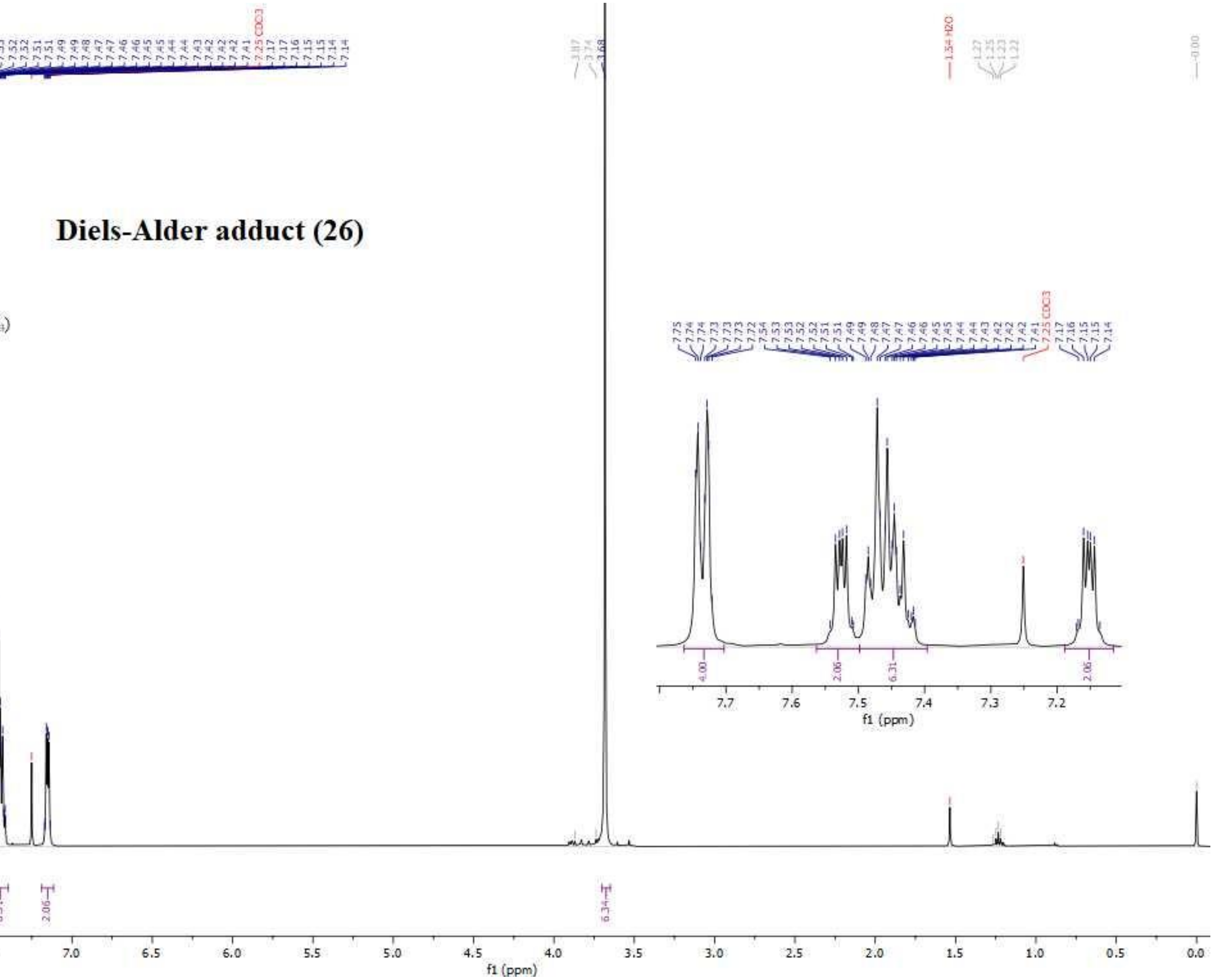

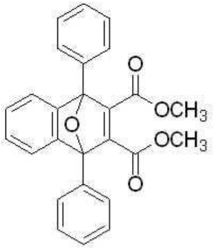

Diels-Alder adduct (26)

^{13}C NMR (126 MHz, $CDCl_3$) δ 164.14, 153.90, 149.13, 133.16, 129.08, 128.58, 128.00, 125.99, 122.20, 94.05, 52.29.

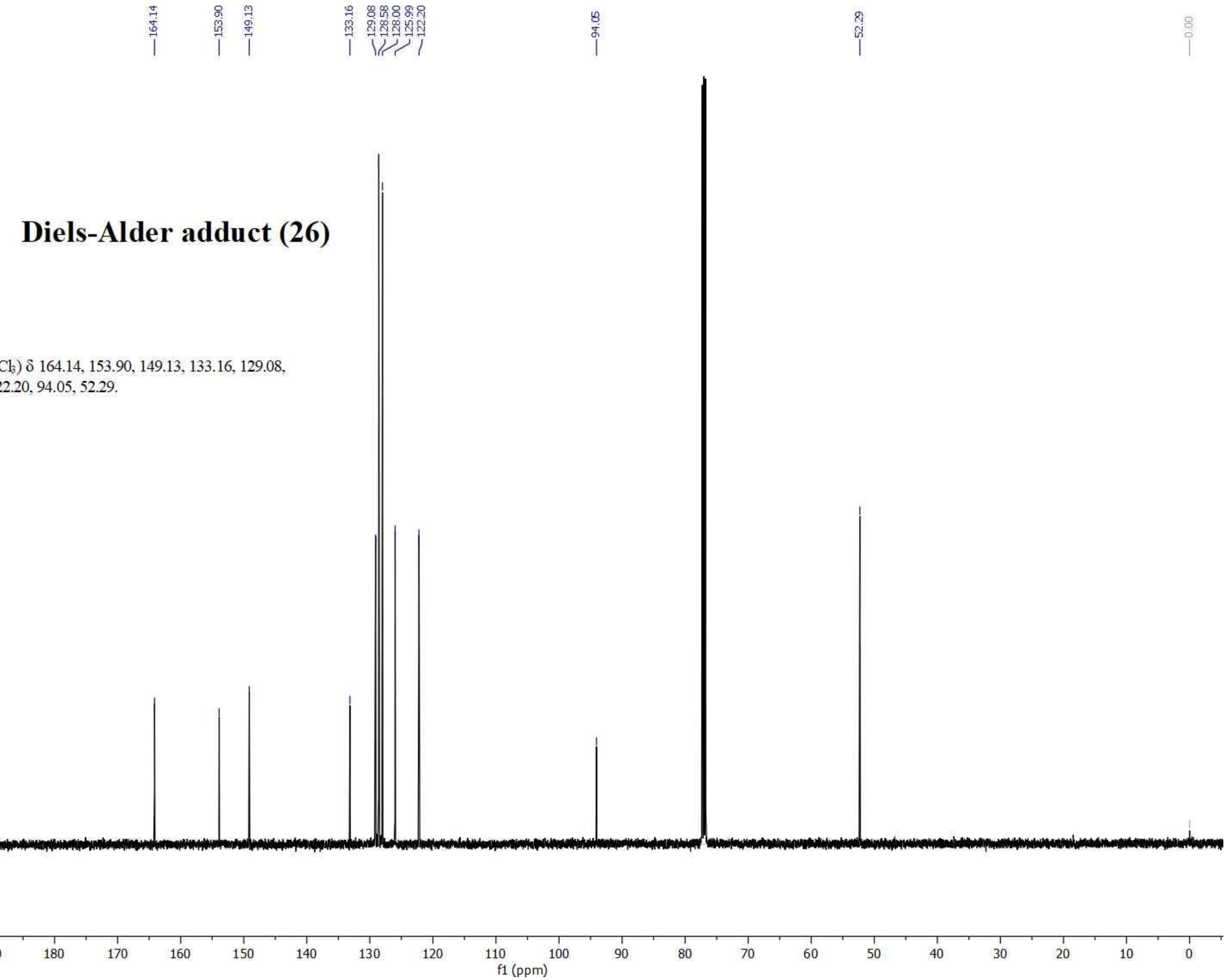

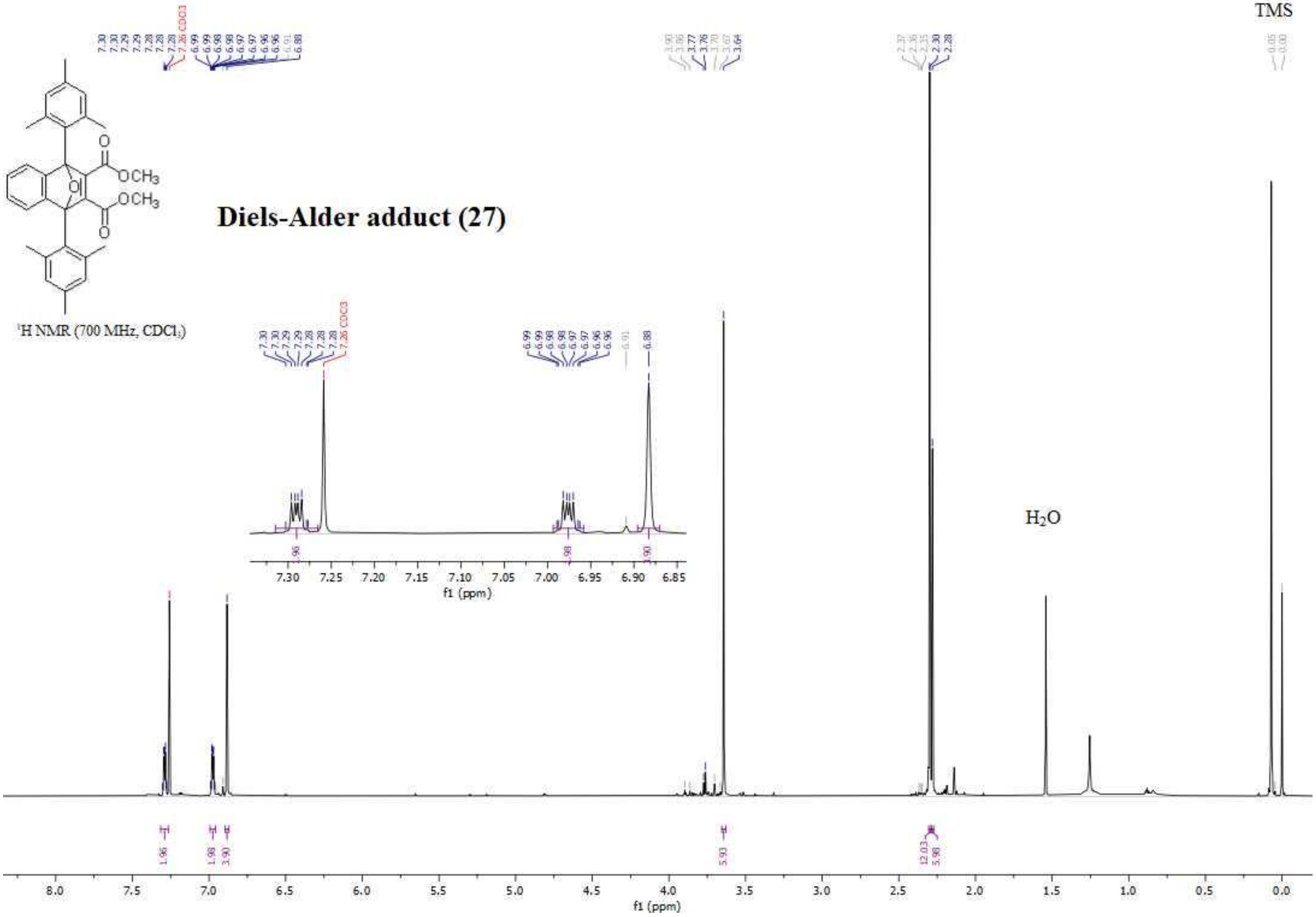

^{1}H NMR (700 MHz, $CDCl_3$)

Diels-Alder adduct (27)

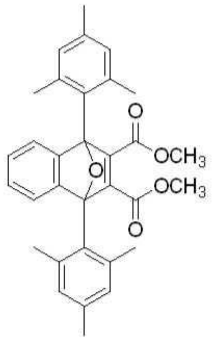

Diels-Alder adduct (27)

^{13}C NMR (176 MHz, $CDCl_3$) δ 164.38, 155.13, 151.10, 139.03, 137.93, 130.68, 128.02, 124.72, 124.49, 95.54, 51.98, 23.71, 20.75.

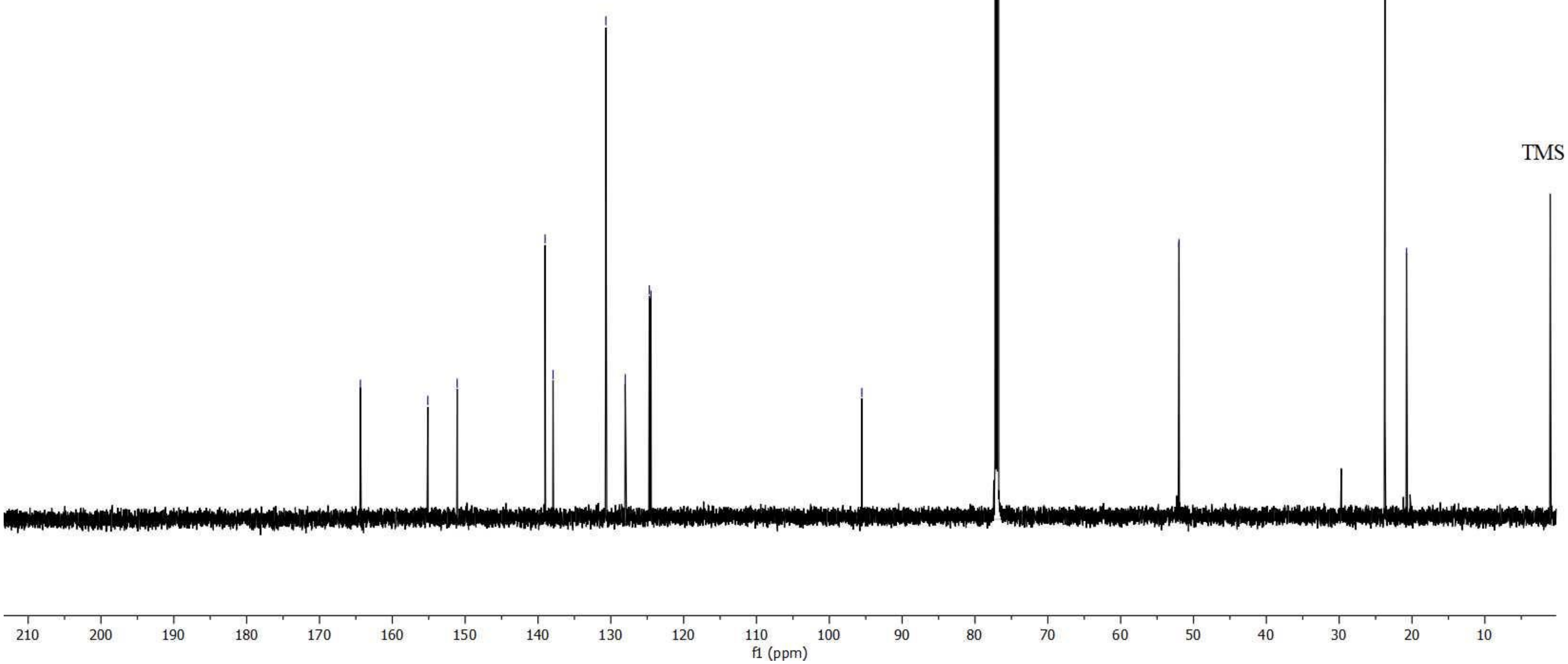

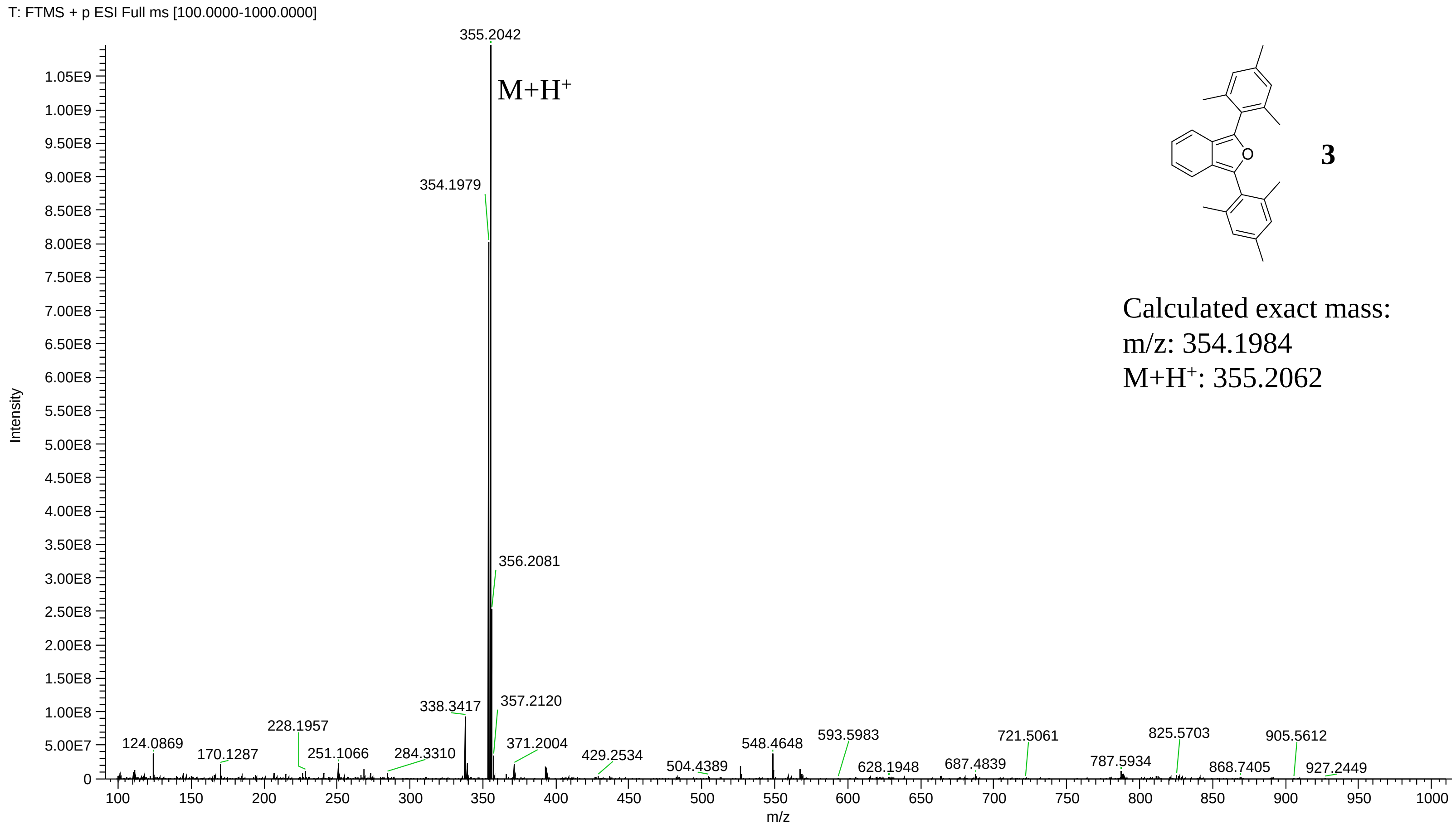

3

Calculated exact mass:
m/z: 354.1984
M+H$^+$: 355.2062

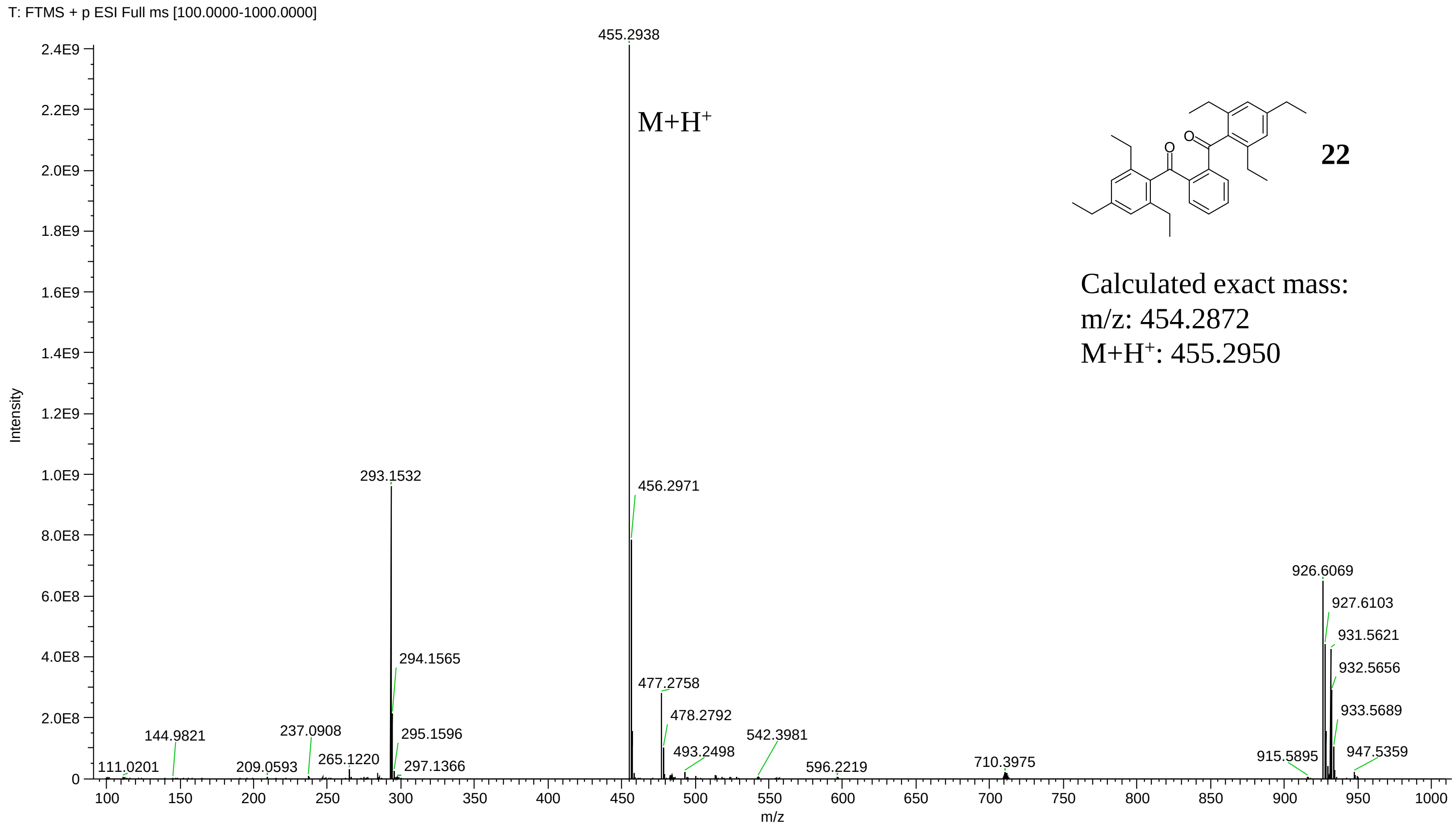

22

Calculated exact mass:
m/z: 454.2872
$M+H^+$: 455.2950

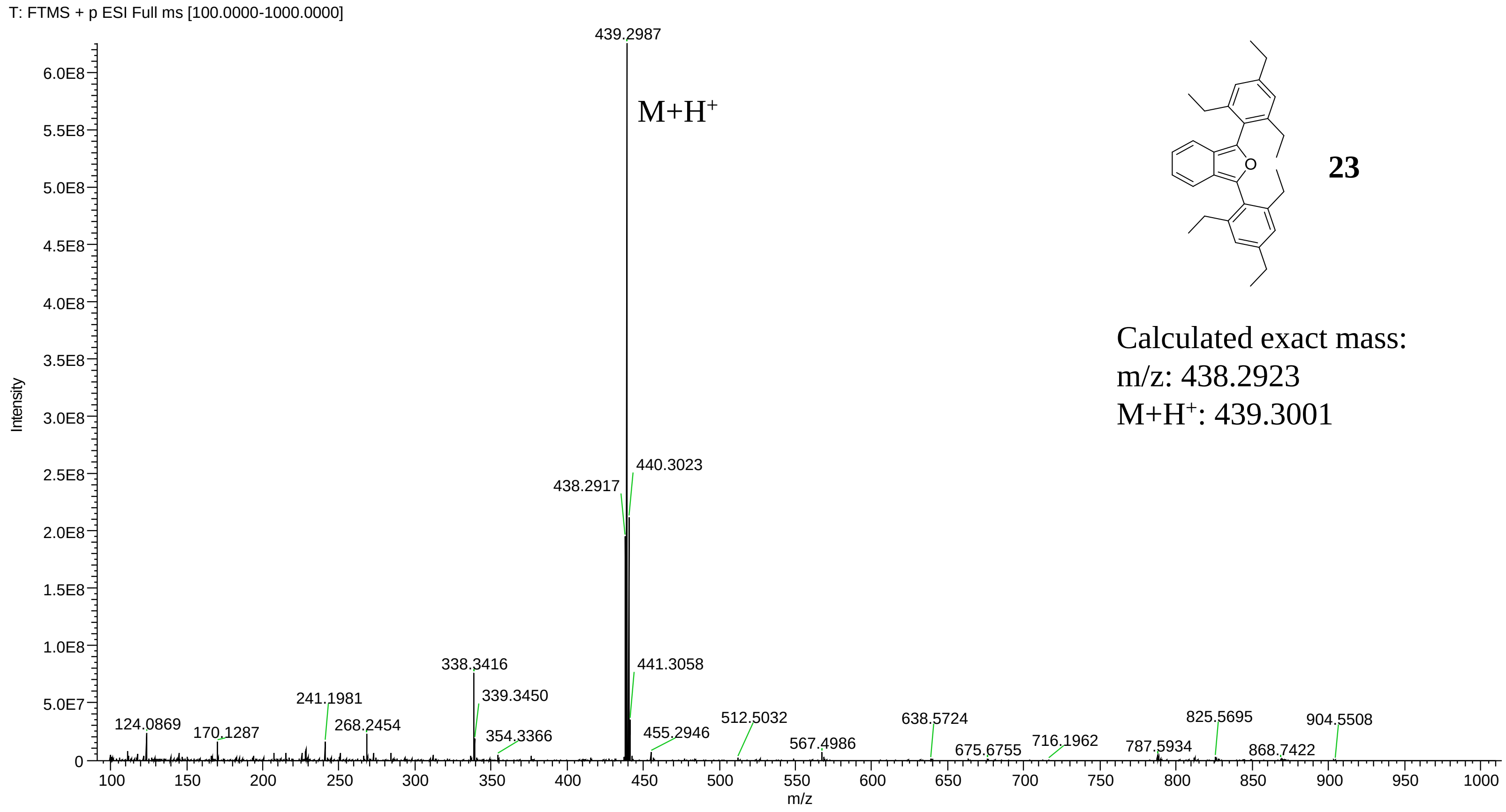

23

Calculated exact mass:
m/z: 438.2923
M+H$^+$: 439.3001

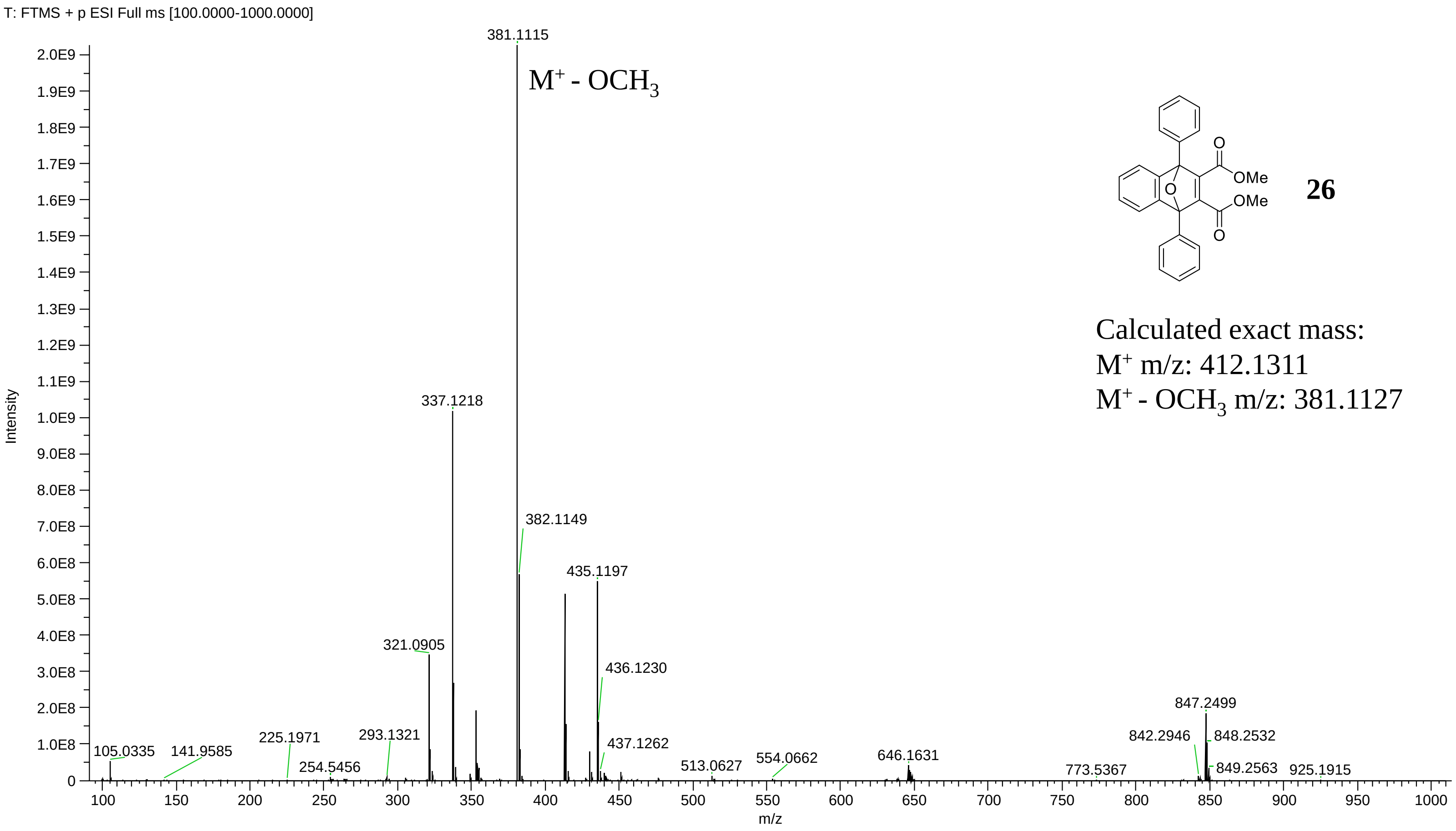

26

Calculated exact mass:
M^+ m/z: 412.1311
$M^+ - OCH_3$ m/z: 381.1127

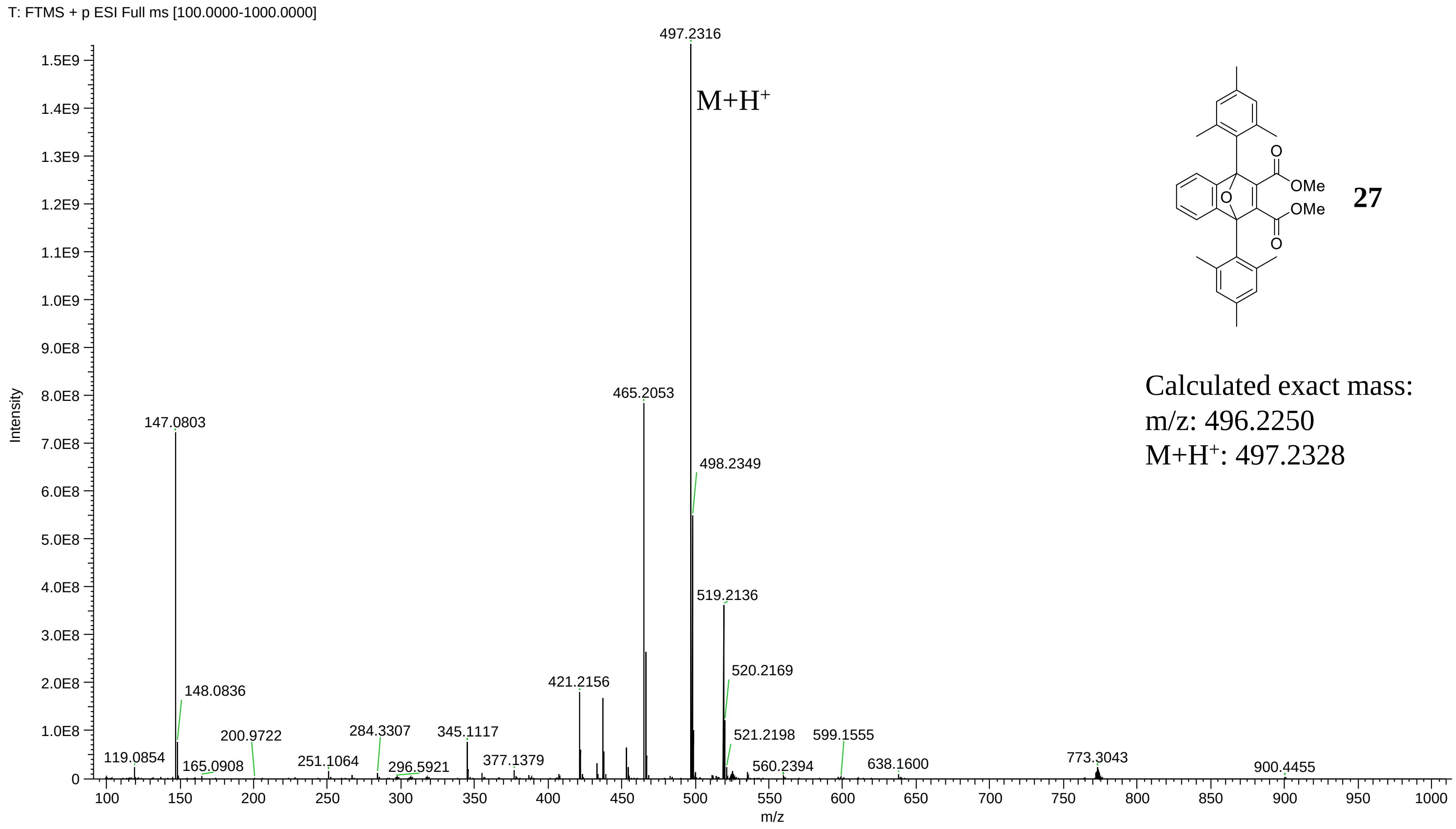

27

Calculated exact mass:
m/z: 496.2250
M+H$^+$: 497.2328
